# Distinct and Convergent Roles of the Synovial Sarcoma X Breakpoint Family of Cancer-Testis Antigens in Ovarian Cancer

**DOI:** 10.3390/ijms27198608

**Published:** 2026-09-26

**Authors:** Kelly C. Grayson, Holly N. Wilkinson, John D. Wright, Stephen J. Archibald, Matthew J. Hardman, Barbara-ann Guinn

**Affiliations:** 1 Centre for Biomedicine, Hull York Medical School, University of Hull, Hull HU6 7RX, UK; john.6.wright@kcl.ac.uk (J.D.W.); stephen.archibald@kcl.ac.uk (S.J.A.); 2 Wound Innovation Institute, University of Hull, Hull HU6 7RX, UK; h.wilkinson@hull.ac.uk (H.N.W.); m.hardman@hull.ac.uk (M.J.H.)

**Keywords:** cancer-testis antigen, ovarian cancer, RNA sequencing, synovial sarcoma X breakpoint, SSX

## Abstract

Ovarian cancer (OC) is a leading cause of death in women, with limited advances in diagnosis and treatment over the past decade. The Synovial Sarcoma, X Breakpoint (SSX) family of cancer-testis antigens represent promising biomarkers and immunotherapeutic targets; however, the functions of individual SSX family members remain poorly defined due to their high sequence similarity. We investigated the roles of SSX2A, SSX2B and SSX3 using RNA sequencing, in vitro and in vivo assays. Pathway analysis revealed involvement in nucleosome assembly, calcium signalling, and cellular stress responses, alongside increased OC cell proliferation in vitro following overexpression. Additionally, SSX variants altered key epithelial–mesenchymal transition (EMT) associated markers, including upregulation of the transcription factor, SLUG. Integration with patient transcriptomic datasets identified several SSX regulated genes associated with overall survival (OS). These findings suggest that SSX family members are not functionally redundant and may contribute to OC progression and clinical outcome.

## 1. Introduction

Ovarian cancer (OC) is the deadliest gynaecological malignancy, largely due to late-stage diagnosis, highlighting the need for improved diagnostics and therapies. OCs are predominately epithelial in origin, with high-grade serous ovarian carcinoma (HGSOC) representing the most common and aggressive subtype [1,2]. Accumulating evidence has shown that HGSOC can originate from serous tubal intraepithelial carcinoma lesions arising within the fallopian tube epithelium [3].

The current clinical biomarker, CA-125, lacks sensitivity in early disease and is influenced by multiple patient factors, limiting its diagnostic utility [4,5,6]. Due to the limitations of current diagnostic techniques, and an unchanging therapeutic landscape, the identification of new markers of OC is essential to improving patient survival. Cancer-testis antigens (CTAs) represent promising biomarkers and immunotherapeutic targets due to their restricted expression in immune-privileged healthy tissues, such as the testis, and elevated levels in cancer cells [7,8].

Members of the Synovial Sarcoma, X Breakpoint (SSX) family belong to a group of predominantly X-linked cancer-testis antigens (CTAs) that were first characterized through their involvement in the SS18-SSX fusion protein found in approximately 95% of synovial sarcoma cases [9]. The SSX family includes nine genes (SSX1-9), with various isoforms caused by splicing, and 10 pseudogenes which have 88–95% nucleotide sequence homology and are clustered primarily within the Xp11 region of the X chromosome with the exception of ψSSX10 [10]. 

SSX2 was identified as the gene encoding the SEREX-defined antigen HOM-MEL-40 [11]. Subsequent RT-PCR analysis has demonstrated variable expression of SSX family members across different cancers, including the detection of SSX4, but not SSX1, SSX2 or SSX3 transcripts, in 6/12 ovarian cancer samples [12]. Sera from epithelial OC (EOC) patients have been shown to contain antibodies against SSX2 and SSX4, while SSX4-specific CD4+ T-cell responses have also been identified, demonstrating that SSX antigens can elicit humoral and cellular immune responses in EOC. [13]. We examined the expression of the SSX antigens in multiple tissue arrays from patients with OC [14,15] and found SSX proteins to be more frequently expressed than indicated by previous RT-PCR analysis by other groups. This likely reflects the non-linear relationship between gene expression and protein levels observed in higher organisms caused by a multi-layered network of post-transcriptional, translational, and degradation mechanisms [16].

Prior studies have often failed to distinguish between SSX family members and even fewer separate the roles of the splice variants, such as SSX2A and SSX2B [17,18,19]. Their high sequence similarity has led to the assumption of functional redundancy, potentially obscuring distinct roles [20]. SSX family members have previously been shown to play roles in cell proliferation, focal adhesion, cell migration and the induction of DNA damage and activation of the p53-p21 pathway [19,21,22]. Chen et al. observed that the overexpression of SSX2 in breast cancer cells increased cell migration and invasiveness, while in prostate cancer cells knockdown of SSX2 increased cell migration, upregulating vimentin while downregulating E- and N-cadherin [19,22]. In contrast, Cronwright et al. showed no effect of SSX knockdown on these genes in melanoma [23]. Although previous studies have explored the functions of SSX variants, systematic comparisons of isoform specific roles within the SSX family have remained lacking until now.

## 2. Results

### 2.1. Engineered SSX Expression Systems Enable Investigation of Isoform Specific Function in OC

The OVCAR-3 cell line was selected as a model of HGSOC based on prior characterisation [24]. Endogenous expression of SSX2A, SSX2B, SSX3 and SSX4 was not observed in OC cell lines, including OVCAR-3, nor the fallopian tube epithelial cell line FT194, making them good model systems for studies of the functional and genetic impacts of SSX expression on these cells (Figure S1). Successful transfection of the target cells was confirmed by detection of the c-Myc tag in vector control (VC) and SSX expression by both western blotting and qPCR for SSX constructs (Figure 1A–I). Peak expression occurred 18–20 h post-transfection (Figure 1A,B).

Validation of the primers and antibodies used in this study was performed utilising OVCAR-3-SSX transient transfectants. The antibody ‘4D10’ detected both SSX2A and SSX2B isoforms (Figure 2A), likely due to sequence overlap at the antibody binding site (Figure S2). This antibody also detected SSX4 (Figure 2A). In contrast, the polyclonal antibody raised against the C terminus of SSX2 (ab48571) detected only SSX2A (Figure 2B). Polyclonal anti-SSX3 antibody ‘SAB1406948’ detected SSX2A, SSX2B and SSX3 (Figure 2C) while the monoclonal SSX4 antibody ‘ab58011’ detected SSX2A, SSX2B, SSX3 and SSX4 (Figure 2D), summarised in Figure 2E. This was a significant limitation of the reagents, and despite numerous attempts to resource other antibodies, none fared any better than the ones chosen for this study. It was not possible to rule out the upregulation of one or more other SSX variants through the transfection of another, or the cross-reactivity of antibodies to the SSX variants.

Validation of SSX qPCR primers also revealed variable specificity and notable cross-reactivity between family members. In SSX2A-overexpressing cells, both SSX2A and SSX2B targeting primers detected transcript expression, whereas in SSX2B-overexpressing cells, strong amplification was observed with the SSX2B primer and minimal detection with the SSX2A primer (Figure 2F,G). Cross-reactivity was most pronounced for the SSX2B primer, which also detected transcripts in SSX3- and SSX4-overexpressing cells, as well as signal with the SSX4 primer in SSX2B cells (Figure 2H,I). This suggests that there were notable cross-reactivities between the primers designed for SSX2B and other SSX transcripts, or that increased SSX transfection of one family member following transfection, caused an increase in transcription of others. Overall, these findings highlight a significant technical limitation in distinguishing SSX isoforms using currently available qPCR primers.

### 2.2. RNA Sequencing Revealed Common and Unique Functions Induced by SSX Variants

RNA-Seq was performed on OVCAR-3 cells overexpressing SSX2A (OVCAR-3-SSX2A), SSX2B (OVCAR-3-SSX2B), SSX3 (OVCAR-3-SSX3) and VC (OVCAR-3-VC). A large proportion of genes (12,341) were commonly expressed across all conditions (A). However, each isoform also induced a distinct subset of differentially expressed genes (DEGs), with 249, 205 and 271 unique genes identified following SSX2A, SSX2B and SSX3, transfection, respectively (Figure 3B–D).

Ingenuity^®^ Pathway Analysis (IPA) software was used to identify significant biological functions and diseases ranked by statistical significance. Gastrointestinal (*p* values = 2.38 × 10^−2^–2.42 × 10^−5^) and hepatic disease (*p* values = 2.38 × 10^−2^–2.42 × 10^−5^) were the top ranked disease pathways that involved genes whose expression was altered by SSX2A transcription (Figure S3). Neither pathway had been previously associated with SSX2A in the literature, although links between SSX2 and hepatic carcinoma have been made [25,26,27]. The most significantly enriched biological functions included cellular function and maintenance (*p* values = 2.38 × 10^−2^–5.32 × 10^−5^), cell-to-cell signalling and interaction (*p* values = 2.38 × 10^−2^–8.95 × 10^−5^), and cell signalling (*p* values = 1.79 × 10^−2^–1.68 × 10^−4^). Further, cancer was identified as one of the most represented categories. Cell signalling networks also had the most involved number of molecules, with 14 identified from the DEG list. IPA was used to highlight the canonical pathways affected (Figure S4) identifying the apelin liver signalling pathway as the most statistically significant and fitting with hepatic system disease being relevant (*p* = 2.76 × 10^−3^) [28].

The Database for Annotation, Visualization and Integrated Discovery (DAVID) [29] identified two functional clusters in the DEGs identified in OVCAR-3-SSX2A cells when compared to OVCAR-3-VC (Table S11). Cluster one included five genes, all of which were categorised as ‘structural constituent of chromatin’ (*p* = 6.5 × 10^−10^). Functionally, this cluster was linked to nucleosome assembly (*p* = 1.9 × 10^−6^), telomere organisation (*p* = 5.6 × 10^−3^) and DNA-replication dependent nucleosome assembly (*p* = 6.6 × 10^−3^). This cluster corroborated previous research that showed SSX2 can impact chromatin structure and function through histone modification [30,31]. Functional characterisation of the second DAVID cluster identified 14 genes associated with membrane-related processes (*p* = 4.9 × 10^−5^). Enriched gene ontology (GO) terms included adenylate cyclase-modulating G-protein coupled receptor signalling (*p* = 3 × 10^−3^), G-protein coupled receptor activity (*p* = 7.4 × 10^−4^), ion transport (*p* = 1.8 × 10^−3^) and transmembrane signalling receptor activity (*p* = 5.2 × 10^−3^). Reactome analysis further identified 89 significantly enriched pathways (*p* ≤ 0.05; Table S12). Histone-related genes including *H2AC6*, *H2BC5*, *H2BC8*, *H3C6* and *H4C12* were present within the most significantly enriched pathways (*p* = 1 × 10^−4^–1.6 × 10^−5^), suggesting a prominent role for chromatin-associated regulatory mechanisms.

To investigate whether SSX2B elicits similar biological effects to SSX2A, DEGs identified in SSX2B-transfected OVCAR-3 cells were subjected to the same IPA workflow. Functional enrichment analysis revealed that cellular, embryonic and organismal developmental processes were among the most significantly enriched categories (Figure S5; *p* = 3.38 × 10^−5^ to 2.67 × 10^−2^). While cellular and organismal development were also significantly enriched in the SSX2A dataset, enrichment of embryonic development appeared to be specific to SSX2B. Cancer-related functions were likewise significantly overrepresented, consistent with the effects observed following SSX2A overexpression (Figure S5, *p* ≤ 0.05). Of the 40 significantly enriched biological function and disease categories identified in SSX2B-expressing cells, 24 were shared with the SSX2A dataset, whereas 16 were unique to SSX2B. These findings suggest that although the two SSX2 isoforms influence a common set of biological processes, they can also regulate distinct functional programmes. Analysis of canonical pathways highlighted further differences between the transcriptional profiles induced by SSX2A and SSX2B. In SSX2B-overexpressing cells, three pathways reached statistical significance: retinoic acid receptor (RAR) activation (*p* = 2.84 × 10^−2^), chondroitin sulphate degradation (*p* = 4.63 × 10^−2^), and dermatan sulphate degradation (*p* = 4.96 × 10^−2^) (Figure S6). Notably, none of these pathways overlapped with those identified following SSX2A overexpression, indicating that the two isoforms may exert their effects through distinct downstream signalling mechanisms.

Two significantly enriched functional clusters were identified following SSX2B overexpression using DAVID; however, their composition differed from those observed in the SSX2A dataset (Table S13). The first cluster was associated with membrane-related components, with the GO term integral component of membrane showing significant enrichment (*p* = 1.2 × 10^−3^). Notably, two genes within this cluster, Otopetrin 2 (*OTOP2*) and reeler domain containing 1 (*REELD1*), were also present in the membrane-associated cluster identified following SSX2A overexpression, suggesting an overlap between the two isoforms. The second cluster was centred on transcriptional regulation and was significantly enriched for genes associated with the GO term regulation of transcription from RNA polymerase II promoter (*p* = 5.0 × 10^−7^). The distinct composition of the DAVID-derived functional clusters supports the notion that SSX2A and SSX2B influence overlapping yet non-identical biological processes. Consistent with observations in the SSX2A dataset, the most significantly enriched Reactome pathways were predominantly associated with chromatin organisation and modification (Table S14). Among these, the histone genes *H2AC6* and *H4C12* were common to both the SSX2A and SSX2B datasets, indicating that modulation of chromatin-related pathways may represent a shared downstream consequence of SSX2 isoform overexpression.

The most significantly enriched functional categories identified by IPA when analysing DEGs following SSX3 overexpression were related to cellular function and maintenance (*p* = 1.66 × 10^−3^ to 4.89 × 10^−2^), cell-to-cell signalling and interaction (*p* = 2.65 × 10^−3^ to 4.89 × 10^−2^), and amino acid metabolism (*p* = 3.33 × 10^−3^ to 6.66 × 10^−2^) (Figure S7). Collectively, these findings suggest that SSX3 influences diverse cellular processes involved in intercellular communication, homeostasis, and metabolic regulation. Comparison of enriched biological functions across SSX family members revealed substantial overlap between isoforms. Of the categories identified following SSX3 overexpression, 31 (77.5%) were also represented in the SSX2A dataset, while 22 (55.0%) overlapped with those identified for SSX2B. Furthermore, 17 functional categories were common to all three datasets, indicating the existence of a shared transcriptional programme associated with SSX family expression. Canonical pathway analysis identified a broader spectrum of significantly enriched pathways in SSX3-overexpressing cells than in either SSX2A- or SSX2B-expressing cells (Figure S8). Among these, calcium signalling emerged as the most significantly enriched pathway (*p* = 5.34 × 10^−3^). In addition, pathways associated with calcium transport were prominently represented and were characterised by a high proportion of upregulated genes. These observations suggest that regulation of calcium homeostasis and signalling may represent a distinctive feature of the cellular response to SSX3 overexpression.

Whilst there were no modified pathways in common with the SSX2B dataset, cAMP response element-binding protein (*CREB*) signalling in neurons, dilated cardiomyopathy signalling and breast cancer regulation by Stathmin1 (*STMN1*) were all found to be commonly altered pathways following transfection of SSX2A and SSX3 (Figures S5 and S9). Overlap of the affected signalling pathways suggested common functions between SSX2A and SSX3 and one functional cluster was identified involving integral components of the membrane (*p* = 7.3 × 10^−9^) (Table S15). When looking at the DEG list translational regulation was identified (*p* = 4.4 × 10^−2^) in contrast to SSX2A and SSX2B, while histones were not found to be part of SSX3’s proposed role. When using Reactome, several functional pathways were identified from the DEG list (Table S16) but like SSX2B, when using adjusted Bonferroni Šidák *p* values, the significance (*p* < 0.05) of these pathways was removed and there was no overlap between the pathways. Although the data suggests that SSX2A, SSX2B and SSX3 have completely independent functions, it is more likely that the differences reflect variations in magnitude and the specificity of transcriptional responses induced by each SSX member.

### 2.3. SSX Overexpression Induces Clinically Relevant Transcriptional Changes in OC Cells

Five DEGs were identified as significantly altered in OC patient samples compared to healthy donor tissue: paraneoplastic antigen MA3 (*PNMA3*), Intestine-specific homeobox (*ISX*), Collectrin (*CLTRN*), Actin Alpha Cardiac Muscle 1 (*ACTC1*) and troponin T (*TNNT2*) (Figure 4A–E). *PNMA3* and *TNNT2* were significantly upregulated in tumours, with a mean FC of 3.27 (*p* = 5.83 × 10^−17^) and 7.29 (*p* = 1.87 × 10^−22^), respectively. In contrast, *ACTC1* and *CLTRN* were significantly downregulated, with mean FC values of −5.26 (*p* = 1.94 × 10^−35^) and −1.14 (*p* = 5.57 × 10^−4^), consistent with their regulation following SSX overexpression in vitro. *ISX* displayed a contrasting pattern, being upregulated following SSX3 overexpression but significantly downregulated in tumour samples (FC = −1.12, *p* = 7.25 × 10^−9^). Additionally, copper metabolism gene MURR1 domain containing 6 (*COMMD6*) and RANBP2-like and GRIP domain containing 8 (*RGPD8*), which were identified as downregulated across all SSX variants using RNA-Seq, were also significantly reduced in OC patient samples (FC = −1.30 and −4.35, respectively; Figure 4F,G). Data for ADP-ribosylation factor-like 17B (*ARL17B*) was not available for comparison.

To assess clinical relevance of the identified DEGs, their association with overall survival (OS) in OC patients was investigated. Elevated expressions of *PNMA3*, *TNNT2* and *ISX* were associated with poorer OS (Figure 4H–J) while increased *CLTRN* expression correlated with a favourable survival outcome and prolonged survival, *p* = 4.7 × 10^−4^ (Figure 4K). Similarly higher *COMMD6* expression was linked to improved survival whilst elevated *RGPD8* was associated with reduced OS (Figure 4L,M). Survival data was not available for *ARL17B* and its prognostic significance could not be assessed.

Based on these survival associations, this subset of DEGs was prioritised for experimental validation by qPCR. *CLTRN*, which was notably downregulated following SSX2B overexpression by RNA-Seq (FC = −21.86, Table S5), was also shown to be downregulated by qPCR, FC = −2.28 (Figure 5A). Analysis of samples transfected with SSX2A, SSX3 and SSX4 also indicated downregulation of *CLTRN* (FC = −0.38, −1.19 and −1.88 respectively). *ACTC1* was shown to be downregulated following overexpression of SSX3 by RNA-Seq (FC = −18.70, Table S6) and showed a consistent trend towards reduced expression by qPCR across SSX variants (Figure 5B). *PNMA3* was shown by RNA-Seq to be upregulated by SSX3 (FC = 22.57, Table S6), which was validated by qPCR (Figure 5C). Transfection with SSX2A and SSX2B also led to an increase in PNMA3 transcripts (FC = 2.47 and 1.48 respectively, Figure 5C). None of the three commonly downregulated genes determined by RNA-Seq, *COMMD6*, *RGPD8* and *ARL17B*, could be validated by qPCR, though *COMMD6* displayed similar trends (Figure 5D–F). In summary, of the genes examined by qPCR, *CLTRN* and *PNMA3* were successfully validated and *ACTC1* and *COMMD6* showed similar trends to that observed in the RNA-Seq data.

However, there were differences in transcript levels observed between the data obtained from RNA-Seq and qPCR, likely reflecting the differences in how these techniques work. RNA-Seq determines relative transcript abundance through sequencing depth, counts mapped digital reads and uses global bioinformatic scaling factors, while qPCR measures target amplification via fluorescence signal kinetics and relies on the ratio between target genes and a reference gene, both of which can vary between biological samples and experimental conditions. These methodological differences may account, at least in part, for the incomplete concordance observed between the data we obtained from RNA-Seq and qPCR.

### 2.4. SSX Overexpression Promotes Proliferative and Migratory Phenotypes and Modulates EMT-Associated Markers

RNA-Seq indicated that the overexpression of SSX2A, SSX2B and SSX3 could modulate biological pathways including ‘cancer’, ‘cellular movement’ and ‘cellular growth and proliferation’ (Figures S3, S5 and S7). In addition, it was observed that SSX2A, SSX3 and SSX4 overexpression significantly increased cell numbers when compared to the VC, whilst SSX2B did not (Figure 6A–E). SSX2A overexpression was shown to be associated with an increase in cell numbers most notably on day six (*p* < 0.05, Figure 6B). SSX3 showed an increase in cell numbers from day three to seven (*p* < 0.05–0.0001, Figure 6D). SSX4 overexpression significantly enhanced cell numbers on day five compared to control cells (*p* < 0.05, Figure 6E). In addition, overexpression of each SSX family member tested promoted cell motility (and/or proliferation) as shown by accelerated wound closure relative to controls. This was most notable following SSX2A and SSX3 overexpression, both of which resulted in significantly greater wound closure rates compared to the VC cells (*p* < 0.05, Figure 6F and Figure S9).

Changes in the transcript levels of known EMT markers were evaluated. SSX2A, SSX2B and SSX3 overexpression led to upregulated SLUG transcripts (Figure 7A–C). SSX3 overexpression increased E-cadherin and vimentin (*p* < 0.0001 and <0.05 respectively, Figure 7D,E).

To investigate the effect of SSX2A, SSX2B, SSX3 or SSX4 overexpression on EMT related genes in early-stage OC, their expression was also examined in the fallopian tube cell line FT194 (Figure S10). A significant increase in SLUG expression was observed following overexpression of SSX3 (*p* < 0.05, Figure 7F). The absence of any observed effects of SSX4 on EMT related markers suggests that SSX4 does not play a role in regulating EMT related molecules in OC, and may have a differing function to SSX2A, SSX2B and SSX3.

SSX3 expression and its association with tumour proliferation was evaluated in a novel xenograft model of OVCAR-3-SSX3, developed in female NOD SCID mice. IHC analysis of excised tumours demonstrated sustained SSX3 overexpression in vivo, with minimal isotype control staining and significantly higher SSX3 expression in OVCAR-3-SSX3 tumours compared to VCs (*p* < 0.001, Figure 7G,H). Ki67 expression was also significantly elevated in OVCAR-3-SSX3 tumours, indicating increased cell proliferation in vivo (*p* < 0.01, Figure 7I).

## 3. Discussion

Previous research from our group has indicated that SSX2 could be a candidate biomarker for early-stage OC [15,32]. Despite this potential, the role of SSX in OC remains elusive. In this study, we investigated the impact of SSX2A, SSX2B, SSX3 and SSX4 on the OC transcriptome. Given the high sequence homology among the SSX family members, antibodies and qPCR primers were validated prior to experimental use to ensure isoform-specific detection.

A key technical finding of this work was the extensive cross-reactivity observed among commercially available antibodies and qPCR primers. Both polyclonal and monoclonal antibodies showed cross-reactivity against multiple members of the SSX family, indicating clonality was not the only consideration when looking at SSX antibody specificity. All antibodies tested, except ab48571, recognised SSX proteins other than their intended targets. Only by targeting the distinct C-terminal region of SSX2A could this isoform be distinguished from SSX2B. This result correlates to that shown by Greve et al., who could distinguish SSX2A from SSX2B, but did not identify an antibody capable of isolating SSX2B [33]. Interpretation of these results is limited by the lack of available epitope information for commercial antibodies. Additionally, cross-reactivity may be influenced by protein folding, as conformational epitopes can arise from discontinuous amino acid sequences [34]. Although no 3D structures are currently available for SSX proteins, their sequence similarity suggests potential structural conservation [35]. However, this is unlikely to impact T cell-based immunotherapies, as intracellular proteins such as SSX are processed into short peptides for MHC class I presentation [36].

While initial qPCR validation suggested potential cross-reactivity between primers, particularly for SSX2B, and raised the possibility that overexpression of one SSX family member might induce expression of others, the subsequent RNA-Seq data did not support this. In the RNA-Seq analysis, both SSX2A and SSX2B appeared on each other’s DEG lists, but neither SSX3 nor other SSX family members were detected as DEGs following SSX3 transfection nor showed increased expression in the raw read counts. Furthermore, in the SSX2A and SSX2B datasets, no other SSX family members were present. These observations suggest that the presence of both SSX2 transcripts is more likely a consequence of sequence similarity affecting read mapping rather than true upregulation of additional family members. Accordingly, the cross-reactivity observed in qPCR likely reflects primer specificity limitations rather than genuine biological co-expression.

It has previously been stated that there is the potential for functional redundancy between SSX family members due to their high sequence similarity [20]. However, the data presented in this study indicated SSX2A, SSX2B and SSX3 each induce a diversity of DEGs and altered biological pathways, and that SSX family members likely perform common and unique functions.

Functional annotation of the SSX2A-regulated transcriptome identified a prominent cluster enriched for histone-related genes, all of which were downregulated following SSX2A overexpression. This observation is notable given previous reports describing interactions between SSX proteins and Polycomb group (PcG) complexes, resulting in altered chromatin organisation and derepression of PcG target genes [30]. Although no components of PcG repressive complexes were differentially expressed in the present dataset, the enrichment of core histone genes supports a possible role for SSX2A in chromatin regulation. Interestingly, the affected genes were restricted to nucleosomal core histones, whereas linker histones were not represented [37]. This pattern may indicate a selective influence of SSX2A on chromatin architecture through modulation of nucleosome-associated proteins. However, because the current RNA-sequencing approach captures transcriptional changes rather than direct protein interactions or epigenetic modifications, the underlying mechanism remains unclear. Consequently, the observed downregulation of histone genes may reflect either indirect transcriptional effects or an unrecognised function of SSX2A that warrants further investigation.

Compared with SSX2A, considerably less is known about the biological activity of SSX3. Given its high sequence homology with SSX2 and conservation of the SSX repression domain (SSXRD), similar interactions with chromatin-regulatory complexes might be anticipated. However, neither the DEGs nor the enrichment analyses identified evidence supporting this hypothesis. Instead, SSX3 overexpression was primarily associated with pathways involved in cellular homeostasis and intercellular communication, with calcium signalling emerging as the most significantly enriched canonical pathway. Dysregulation of calcium signalling is recognised as an important contributor to tumour progression, influencing proliferation, migration, invasion, and cell survival [38]. SSX3 overexpression was shown to be associated with increased expression of *CAMK1*, a calcium/calmodulin-dependent serine/threonine kinase implicated in multiple malignancies. Elevated *CAMK1* expression has been reported in breast and renal cancers and has been associated with adverse clinical outcomes in acute myeloid leukaemia [39,40,41]. Furthermore, increased *CAMK1* expression has been observed in EOC and linked to reduced patient survival [42]. Taken together, these findings suggest that SSX3 may promote oncogenic signalling through modulation of calcium-dependent pathways, potentially contributing to tumour progression and poorer clinical outcomes. Nevertheless, mechanistic studies will be required to determine whether *CAMK1* represents a direct downstream target of SSX3 or is altered as part of a broader transcriptional programme.

The integration of RNA-Seq derived data with patient survival analyses revealed some relationships between transcriptional changes induced by SSX overexpression and clinical outcomes in patients. Notably, several genes that were upregulated in RNA-Seq datasets, such as *PNMA3* and *TNNT2*, were also associated with poorer OS when highly expressed in patient samples, suggesting that SSX-driven transcriptional programs may promote more aggressive disease phenotypes. Conversely, genes that were downregulated following SSX overexpression, including *CLTRN* and *COMMD6*, were linked to improved survival when expressed at higher levels, supporting a potential tumour-suppressive role that is diminished in the presence of SSX activity. Collectively, these findings suggest that the directionality of gene expression observed by RNA-Seq broadly aligned with their prognostic significance. Of the three genes found to be downregulated across all datasets (*COMMD6*, *RGPD8* and *ARL17B*), a plot could not be generated for *ARL17B*. Missing information due to alternative splicing has been recognised as a limitation of this database by the authors [43].

Previous research has shown that SSX2 is involved in promoting the migration and invasion of malignancies including breast cancer and melanoma [19,23]. In prostate cancer SSX2 was associated with genes linked to EMT and metastasis and shown to increase migratory capacity [22]. Analysis of RNA-Seq data highlighted ‘cellular movement’ and ‘cellular growth and proliferation’ as modulated pathways in OC following overexpression of SSX2A, SSX2B and SSX3. Additional enriched categories, including ‘cell signalling’, ‘cell cycle’ and ‘cancer’, further support a role for SSX in regulating processes central to cell growth, differentiation, motility and invasiveness. Consistent with these observations, overexpression of SSX family members was associated with enhanced proliferative and/or metabolic activity in OVCAR-3 cells through the MTS assays as well as their increased proliferative/migratory capabilities in wound healing assays.

Many CTAs have been implicated in tumour progression, including in the acquisition of invasive and metastatic phenotypes. CTAs such as NY-ESO-1 and members of the MAGE family have been reported to enhance the aggressiveness of OC cells [44,45]. Although the contribution of SSX proteins to the EMT process remains incompletely defined, existing evidence suggests their role in regulating cellular plasticity. In melanoma models, suppression of SSX expression reduced *MMP2* levels and was associated with a shift towards a mesenchymal-to-epithelial transition phenotype [23]. Furthermore, interactions between SSX family proteins and β-catenin have linked SSX expression to Wnt signalling, a key regulator of EMT and metastatic dissemination [46]. In this study, the overexpression of SSX2A, SSX2B, and SSX3 consistently increased expression of the EMT-associated transcription factor *SNAI2* (SLUG). SLUG is a well-established driver of tumour progression and has been associated with enhanced invasion, metastasis, and therapeutic resistance across multiple malignancies [47,48]. In OC, elevated SLUG expression has been correlated with advanced tumour grade and metastatic disease [49]. These findings support a role for SSX family members in promoting transcriptional programmes associated with a more aggressive tumour phenotype. Notably, SSX3 overexpression was found to increase the expression of both *CDH1* (E-cadherin) and *VIM* (vimentin). While upregulation of vimentin is consistent with enhanced cellular motility and regulating EMT-related molecules, increased E-cadherin expression is traditionally associated with maintenance of an epithelial phenotype [50,51]. This apparent discordance may reflect the complexity of epithelial-mesenchymal plasticity, whereby tumour cells can simultaneously express epithelial and mesenchymal markers and adopt hybrid phenotypic states that facilitate metastatic progression. Emerging evidence suggests that the role of E-cadherin in OC may differ from that observed in other epithelial malignancies. For example, increased *CDH1* expression has been reported in OC ascites compared with matched primary tumours and was associated with higher CA-125 concentrations and shorter disease-free survival [52]. These observations indicate that E-cadherin expression does not necessarily signify a less aggressive phenotype in OC and may instead contribute to tumour cell survival and dissemination within the peritoneal cavity. Consequently, the concurrent upregulation of E-cadherin and vimentin observed following SSX3 overexpression may reflect a transition towards a more plastic cellular state with enhanced metastatic potential.

Contrary to the results shown in OVCAR-3, SSX2A, SSX2B and SSX4 did not alter any of the investigated EMT markers significantly in the fallopian tube cell line, FT194. However, overexpression of SSX3 in FT194 led to a significant upregulation of SLUG expression. The increase in SLUG expression in both the late (OVCAR-3) and early (FT194) OC models following SSX3 transfection suggested that SSX3 played a role in SLUG mediated signalling pathways that could include EMT. However, all studies were performed shortly after gene transfection and stable transfectants may show different results.

To investigate the role of SSX3 in cell proliferation further, expression of the cell proliferation marker Ki67 was assessed by IHC. A statistically significant increase in Ki67 levels was detected in tumour xenografts overexpressing SSX3 in comparison to the VC, therefore demonstrating that SSX3 expression impacts OC proliferation in vivo.

Despite these insights, limitations of the study should be considered. All functional experiments were performed using transient overexpression systems in cell lines lacking endogenous SSX expression, which may not fully recapitulate physiological conditions. In addition, this study restricted its scope to SSX2–4 and therefore did not evaluate the potential contributions of other family members. Only some DEGs had their expression validated by qPCR. Those chosen were determined by RNA-Seq data and availability of appropriate primers. Low serum increased the likelihood that the wound healing assay was measuring migration rather than proliferation but low serum can introduce major physiological stress and lead to unpredictable changes in cell signalling, behaviour and cell death over time (as what little serum is available becomes depleted). The MTS assay can also measure cellular metabolic activity rather than just direct cell viability. The in vivo studies of the impact of SSX3 on OVCAR-3 cells were limited to one SSX family member and therefore, we cannot conclude that all SSX family members are in vivo regulators of OC proliferation in general. Nevertheless, this study establishes a basis for the differing and overlapping functional roles of each SSX family member tested here and supports the value of further exploration of their roles in OC biology.

## 4. Materials and Methods

### 4.1. Cell Culture

OVCAR-3 cells (CVCL_0465) and FT194 cells (CVCL_UH58) were purchased from American Type Culture Collection (ATCC, Manassas, VA, USA). Culture conditions and cell line characteristics can be found in Table S1. Cells were kept at 37 °C and 5% CO_2_ in a 95% humidified incubator. Routine mycoplasma testing was performed using the EZ-PCR Mycoplasma Detection Kit (Sartorius, Epsom, UK).

### 4.2. Overexpression of SSX Family Members

pCMV6-Entry plasmids encoding SSX2A, SSX2B, SSX3 or SSX4 were purchased from OriGene, Rockville, MD, USA. The pCMV6-Entry VC was kindly donated by Professor A Banham, University of Oxford. Cells were transfected according to the manufacturer’s instructions. Briefly, 2–4 × 10^5^ cells/mL were seeded in 60 mm dishes and incubated for 24–48 h until the cells reached 70–90% confluence. Spent media was removed and the cells were washed in PBS. A transfection mixture containing Lipofectamine 2000 (Thermo Scientific, MA, USA) and 5.5 µg plasmid DNA were combined in serum free media (Thermo Scientific, Waltham, MA, USA) and incubated for 10 min at RT to form DNA–lipid complexes. Following the addition of 5 mL OptiMEM reduced serum media (Thermo Scientific, Waltham, MA, USA), the transfection mixture was added to the cells and incubated with gentle rocking for 4 h. The transfection mixture was removed and complete growth media added. Cells were then collected for gene and protein analysis following optimized post-transfection periods of 18 h.

### 4.3. RNA Isolation and cDNA Synthesis

RNA was extracted from OVCAR-3 cells overexpressing SSX2A, SSX2B, SSX3, SSX4 or the VC using the RNeasy^®^ Mini Kit (Qiagen, Hilden, Germany) following the manufacturer’s instructions, and all data are representative of three experimental repeats. Samples were quantified using a Nanodrop Light and stored aliquoted at −80 °C until required. First strand cDNA synthesis was performed from each sample using the RevertAid First Strand cDNA Synthesis Kit (Thermo Scientific, Waltham, MA, USA) as follows: −1 µg RNA was diluted in 11 µL of nuclease free water. A master mix containing 5 X Reaction Buffer, 20 U/µL RiboLock RNase inhibitor, 10 mM dNTP mix, 200 U/µL RevertAid M-MuLV Reverse transcriptase and 100 µM Random Hexamer primer in a final volume of 20 µL. Samples were mixed and placed in a thermocycler (Bio-Rad, Hercules, CA, USA) for 5 min at 25 °C, 1 h at 42 °C and 5 min at 70 °C. cDNA was aliquoted and stored at −20 °C until required.

### 4.4. RNA Sequencing

Transcriptomic profiling was performed by Novogene (Cambridge, UK) using the Illumina Novaseq platform on RNA generated during three independent biological experiments. Sequencing quality was high across all libraries with base error rates below <0.3% and >97.5% of reads passing quality filtering. Subsequently, the reads were mapped to the reference genome using HISAT2 (v2.0.5). Gene-level quantification was performed using FeatureCounts software (v1.5.0-p3) after transcript assignment. Gene expression was normalised using the fragments per kilobase of transcript sequence per million base pairs sequenced (FPKM) method. DESeq2 R package (1.20.0) was used to perform differential gene expression analysis utilizing the Benjamini and Hochberg’s method to adjust *p*-values and limit false discovery rates. Genes that met the criteria of ±1.5 fold change (FC) between the test and control sample and had a *p* ≤ 0.05 were classified as DEGs. The R Bioconductor package ‘EnhancedVolcano’ [53] was used to generate Volcano plots and the functional analysis of DEGs was performed using DAVID. Enriched signalling pathways were identified using IPA software (Qiagen, Hilden, Germany).

### 4.5. OC Patient Transcriptomic Dataset Analysis

The most statistically significant DEGs were analysed in OC patient transcriptomic datasets from The Cancer Genome Atlas (TCGA), Therapeutically Applicable Research to Generate Effective Treatments (TARGET), and the Genotype-Tissue Expression databases [43] using TNM plotter ([54]; TNMplot). A total of 133 healthy donors ovarian and 374 primary OC tissues with cutoffs of Mann–Whitney *p* < 0.05 and expression in more than 10 tumour and/or healthy tissues were considered. Kaplan–Meier plotter ([55]; https://kmplot.com/, accessed 1 July 2023) data used Cox proportional hazards regression and the computation of the False Discovery Rate to determine the OS of OC patients (*n* = 374) according to the level of DEG mRNA expression within cohorts assigned according to the best cutoff level with statistical analysis performed using Logrank.

### 4.6. Quantitative Real-Time qPCR (qRT-PCR)

Real-time quantitative PCR (qPCR) was performed on each sample in triplicate using QuantiNova™ SYBR^®^ Green PCR kit with QuantiTECT primer assays (Table S2; Qiagen, Hilden, Germany) according to the manufacturer’s instructions. Briefly, 5 ng of cDNA diluted in diethylpyrocarbonate (DEPC; Fisher Scientific, Loughborough, UK)-treated water was added to a mastermix containing QuantiNova SYBR Green PCR Master Mix, the appropriate primer and RNase-free water to a final volume of 100 µL per well. Non-template controls were prepared using RNase-free water with no cDNA. All samples were analysed in triplicate in a MicroAmp^®^ Fast Optical 96-well reaction plate sealed with optical adhesive film (Thermo Scientific, USA). GAPDH was used as the reference gene. A StepOnePlus Real-time PCR System (Thermo Scientific, Waltham, MA, USA) was used for the cycling program as follows: −2 min at 95 °C, 35–40 cycles of [5 s at 95 °C and 10 s at 60 °C], followed by 15 s at 95 °C, 1 min at 60 °C, and 15 s at 95 °C for the melt curve. Data was exported from StepOne Plus software (Thermo Scientific, Waltham, MA, USA) for analysis of the fold change in the gene expression relative to the VC using the 2^−^^ΔΔCT^ method [56].

### 4.7. Polyacrylamide Gel Electrophoresis and Western Blotting

Confluent layers of adherent cells were washed in 1 X PBS and harvested using TrypLE Express (ThermoFisher). Cells were washed in PBS and pelleted at 20,000× *g*. Supernatant was removed and the cell pellet resuspended in 50–200 µL UTB lysis buffer (9 M Urea, 75 mM Tris-HCl, 0.15 M β-mercaptoethanol) depending on the size of the pellet. The cells were sonicated (Diagenode, Seraing, Belgium) for 5 min on a high setting and centrifuged for 20,000× *g* for 15 min at 4 °C. The supernatant was transferred to a clean Eppendorf tube, quantified using a Nanodrop Light against UTB buffer alone and diluted to obtain 30 µg in 7.5 µL and 7.5 µL of 2 × Laemmli sample buffer (3.3% sodium dodecyl sulfate, 6 M urea, 17 mM Tris-HCl pH 7.5, 0.07 M β-mercaptoethanol and 0.01% bromophenol blue) was added. Samples were heated at 95 °C for 5 min, placed on ice, microfuged briefly and then separated using 8–15% polyacrylamide gels at 100–120 V for 1–2 h until the migration front reached the bottom of the gel. A total of 5 µL of Precision Plus Protein Dual Colour Standard (Bio-Rad, Hercules, CA, USA) was loaded into 1 well. Proteins were transferred to 0.45 μm polyvinylidene difluoride (Merck, Darmstadt, Germany) membranes and immunoblotted with primary antibodies (Table S3) and HRP-conjugated secondary antibodies (goat anti-rabbit or anti-mouse IgG H&L (Abcam, Cambridge, UK) were used. Antibody bound proteins were visualised using enhanced chemiluminescence solution (GE Healthcare, Chicago, IL, USA) with the ChemiDoc XRS+ System (Bio-Rad, Hercules, CA, USA).

### 4.8. Cell Proliferation

Cellular viability was determined using CellTiter 96^®^ AQ_ueous_ One Solution Cell Proliferation Assay (Promega, Madison, WI, USA) according to manufacturer’s instructions. Briefly, the CellTiter 96^®^ AQ_ueous_ One Solution contains a tetrazolium compound [3-(4,5-dimethylthiazol-2-yl)-5-(3-carboxymethoxyphenyl)-2-(4-sulfophenyl)-2H-tetrazolium, inner salt; MTS] and an electron coupling reagent (phenazine ethosulfate; PES). Cells were seeded in complete media at 2 × 10^3^ cells per 100 µL in triplicate in a 96-well clear bottom plate (Sarstedt, Nümbrecht-Rommelsdorf, Germany). Cells were incubated at 37 °C for 1–7 days with media replaced every third day. A total of 20 µL of CellTiter 96^®^ AQ_ueous_ One Solution was added to each well for 2 h at 37 °C, prior to the time point of interest. The quantity of formazan produced was measured at 490 nm on an Absorbance Microplate Reader (ELx800, Agilent Technologies, Santa Clara, CA, USA). Statistical analysis was performed using a two-way ANOVA with Šidák correction.

### 4.9. Wound Healing/Migration Assay

Cells were seeded at 2 × 10^6^ per well in triplicate in 6-well plates. After 24 h, a scratch was made down the centre of the well using a sterile 20 µL pipette tip, disrupting the cell monolayer. Low serum media (0.5% FBS, Thermo Scientific, MA, USA) was added to minimise cell proliferation so that gap closure instead reflects cell migration [57]. The scratch was visualised immediately after making the scratch (T = 0) and after 24 h (T = 24) using a Zeiss Axio Inverted Fluorescence microscope (Zeiss, Jena, Germany). Scratch closure was calculated using ImageJ (version 1.53) with the ‘wound healing size tool’ plugin [58,59]. The wound closure % was calculated using the following formula where *A* is the area of the wound:

$$Wound\;Closure\;\%=(\frac{At=0-At=24}{At=0})\times 100\%$$

Statistical analysis was performed using a one-way ANOVA with Dunnett correction.

### 4.10. Animal Experimentation

Animal experiments were approved by the University of Hull Animal Welfare Ethical Review Body, approval code P8D22271F, SA28 (approval date 19 December 2019) and performed in accordance with the UK Animals (Scientific Procedures) Act 1986 and the associated Code of Practise for the care and use of protected animals in scientific procedures as well as the guidance of the National Cancer Research Institute Committee on the Welfare of Animals in Cancer Research [60] under Home Office Project License number P8D22271F.

Female non-obese diabetic severe combined immunodeficient (NOD SCID) mice (6 weeks old; Janvier Labs, Le Genest-Saint-Isle, France) were maintained under standard housing conditions with controlled temperature and humidity, a 12 h light/dark cycle and ad libitum access to food and water. Mice were injected subcutaneously into the right shoulder under anaesthesia with 200 µL total volume consisting of 100 µL of either 10^6^ OVCAR-3-VC or OVCAR-3-SSX3 viable cells in PBS and 100 µL Matrigel^®^ (Corning, Corning, NY, USA) (*n* = 5 or 4 per group respectively).

### 4.11. Histology

Excised tumour tissues were fixed overnight in preservative containing 40 mL formaldehyde, 10 mL glacial acetic acid (Thermo Scientific, Waltham, MA, USA), 4.5 g sodium chloride (Thermo Scientific, Waltham, MA, USA), 0.25 g alkyltrimethylammonium bromide (Sigma-Aldrich, MO, USA) in 450 mL dH_2_O. Following fixation, tumour tissue was transferred into 70% ethanol for at least 24 h prior to paraffin embedding. Paraffin blocks were sectioned at 5 µm thickness using the Leica RM225 microtome (Leica Camera, Wetzlar, Germany) and mounted onto VECTABOND^®^ (Vector Laboratories, Newark, CA, USA) treated microscope slides. Before, immunolabelling slides were de-paraffinised in 100% xylene for 30 min and rehydrated through a graded ethanol series consisting of two washes in 100% ethanol followed by 90% ethanol, 70% ethanol and 50% ethanol for 2 min each. Slides were thoroughly rinsed in dH_2_O.

Antigen retrieval conditions were optimised according to the target antigen. For Ki67 immunolabelling (Table S3), tissue sections were boiled in sodium citrate buffer for 10 min, allowed to cool for 10 min and placed in 1X PBS for a further 15 min. For all other antigens, tissue sections were subjected to heat-mediated retrieval in sodium citrate buffer for 2–3 min followed by a 10 min cooling period. Endogenous peroxidase activity was quenched by a 20 min incubation in 0.3% H_2_O_2_ in dH_2_O (Sigma-Aldrich, Burlington, MA, USA) prior to a 1X PBS rinse for 15 min. For immunolabelling, VECTASTAIN^®^ Elite^®^ ABC kit (Vector Laboratories, Newark, CA, USA) was used in accordance with manufacturer’s instructions and immunolabelled tissue sections were imaged using a Nikon Eclipse E400 microscope (Nikon, Tokyo, Japan). Isotype controls of MOPC-21 (Abcam; ab18443), a monoclonal mouse anti-human antibody and a polyclonal rabbit antibody (Ab37415) were used as negative controls in immunolabelling experiments. Anti-β-actin (ACTN05) was used as a positive control. All antibodies were used at a concentration of 1:100 for immunolabelling and 1:1000 for Western blotting.

### 4.12. Haematoxylin and Eosin Staining

Tumour morphology was examined following differential staining using a 15–95 s incubation in haematoxylin (Thermo Scientific, Waltham, MA, USA), a rinse in excess dH_2_O, and an 1–2 s incubation in 0.1% Eosin B in dH_2_O (Sigma-Aldrich, Burlington, MA, USA). Tissue was immediately removed from the stain and rinsed in excess dH_2_O.

### 4.13. Histology Scoring

Immunolabelling was evaluated using a modified semi-quantitative Quickscore approach [61]. Staining intensity was graded on a four-point scale (0, no staining; 1, weak; 2, moderate; 3, strong) while the proportion of immunolabelled cells were scored as a percentage of each possible intensity: 0, <25%; 1, 25–50%; 2, 50–75%; 3, >75%. An overall immunoreactivity score was calculated by multiplying the intensity and proportion scores, generating a range of values from 0 to 12. Final scores were categorized as 0–1, absent/background; 2–3, weak; 4–8, moderate; 9–12, strong. All tissue scoring was performed in a blinded manner with samples analysed in a random order and without access to the experimental group information.

### 4.14. Statistical Analyses

Statistical analyses were performed using two-tailed student’s *t*-tests and one- or two-way ANOVA in GraphPad Prism 7 (GraphPad Software Inc., San Diego, CA, USA).

## 5. Conclusions

In summary, this work challenges the assumption of functional redundancy within the SSX family and demonstrates that individual isoforms may exert both shared and distinct effects on gene expression and cellular phenotype. These findings provide a foundation for further investigation into the role of SSX proteins in OC and support their potential as biomarkers and therapeutic targets.

## Figures and Tables

**Figure 1:**
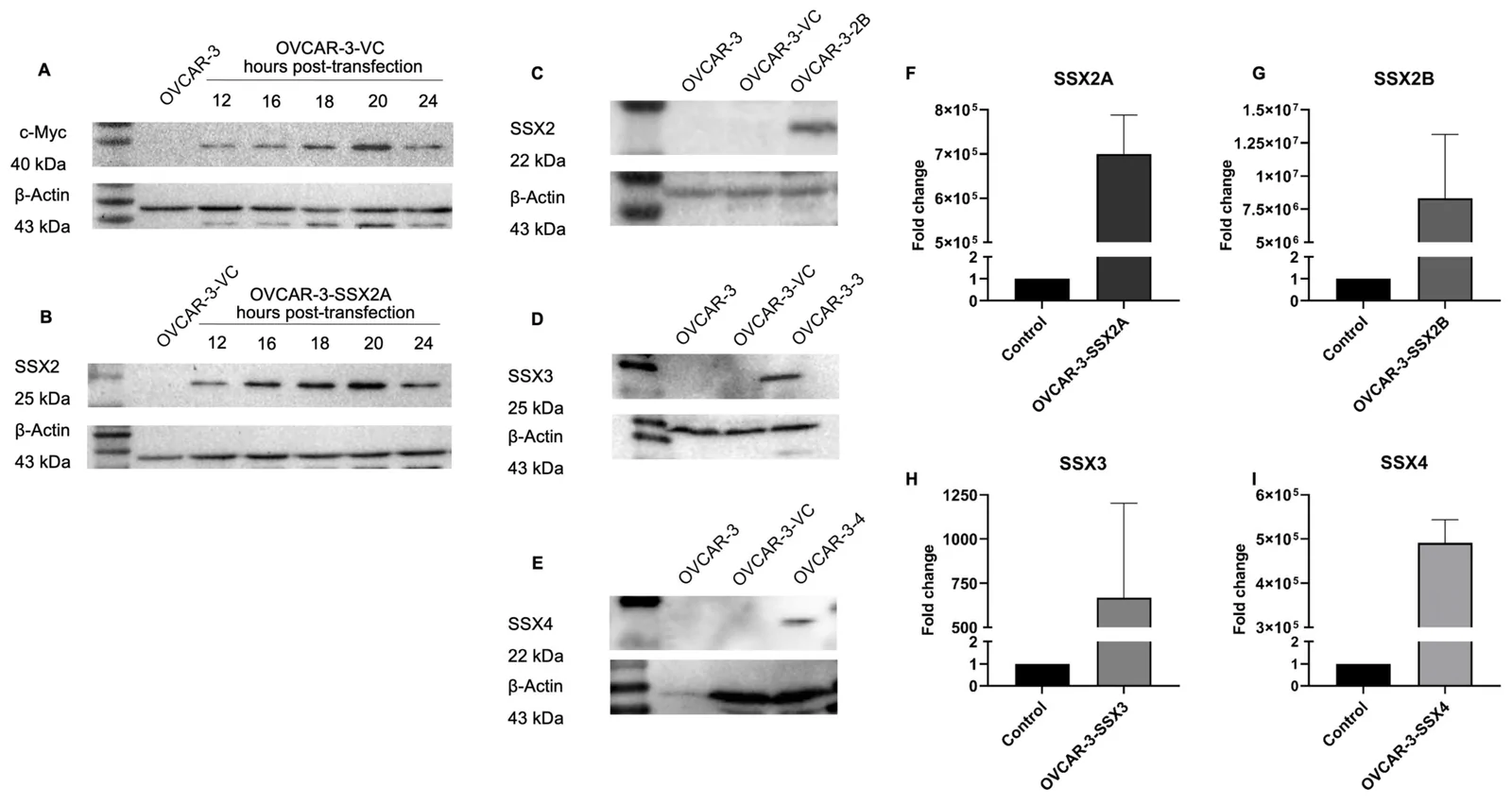
Generation of SSX2A, SSX2B, SSX3 and SSX4 overexpression models in OVCAR-3. (**A**) c-myc expression in OVCAR-3-VC cells compared with (**B**) SSX expression in OVCAR-3-2A, both of which were shown to peak between 18–20 h post-transfection. OVCAR-3 cells were transiently transfected with (**C**) SSX2B, (**D**) SSX3 and (**E**) SSX4. Confirmation of transfection with (**F**) SSX2A, (**G**) SSX2B, (**H**) SSX3 or (**I**) SSX4 relative to expression of OVCAR-3 transfected with a VC plasmid by qPCR. The fold change was calculated relative to the VC using the 2^−ΔΔCT^ method. GAPDH was used as the endogenous reference gene. Data representative of *n* = 3 experimental repeats, error bars ± SEM.

**Figure 2:**
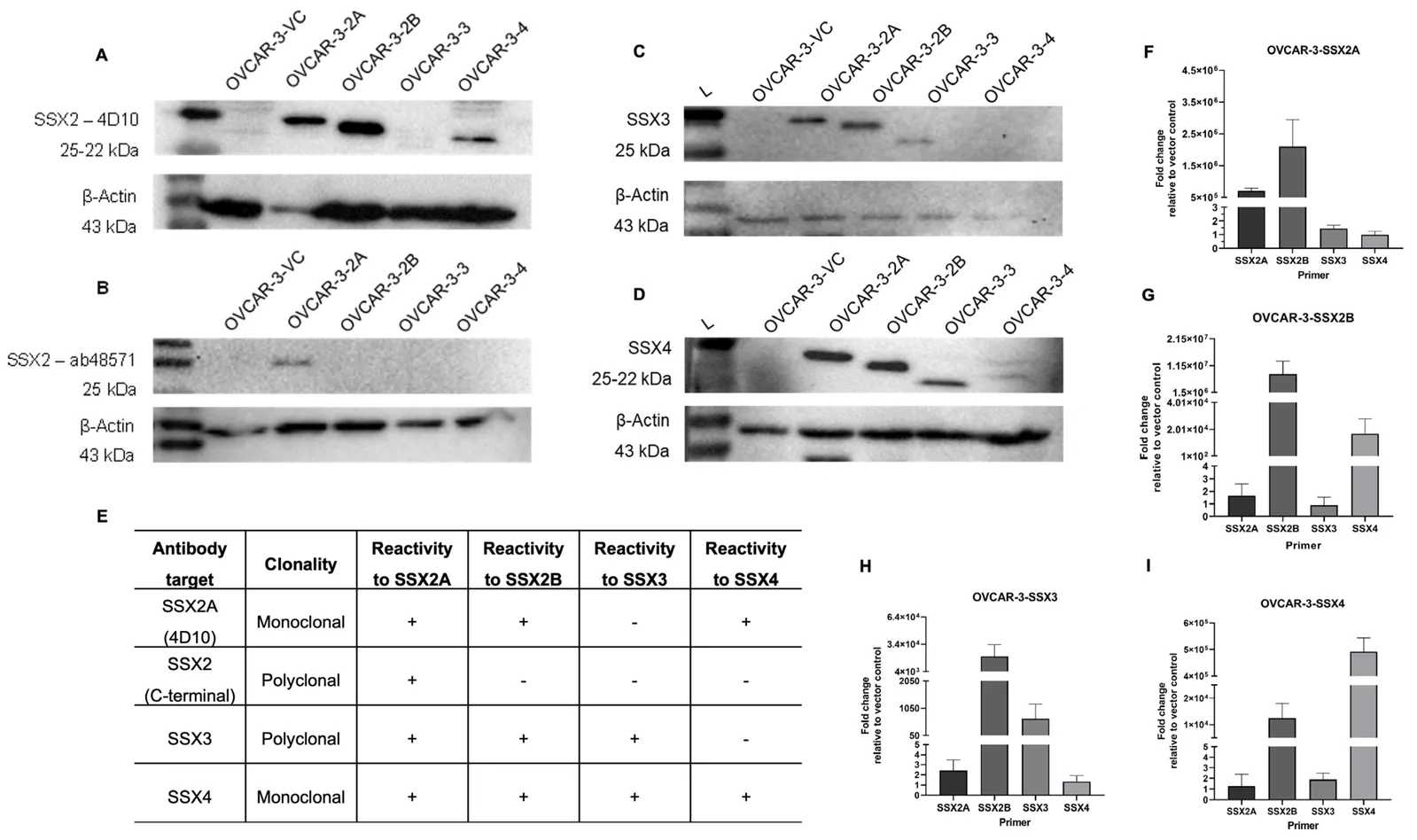
Antibodies to SSX family members show cross-reactivity. (**A**) Monoclonal SSX2 antibody ‘4D10’ identified SSX2A, SSX2B and SSX4 expression. (**B**) Polyclonal SSX2 antibody ‘ab48571’ was able to detect only SSX2A expression. (**C**) Polyclonal SSX3 antibody ‘SAB1406948’ indicated SSX2A, SSX2B and SSX3. (**D**) Monoclonal SSX4 antibody ‘ab58011’ indicated SSX2A, SSX2B, SSX3 and SSX4 expression. (**E**) Summary of the cross-reactivity of commercially available SSX antibodies. (**F**) SSX2A transcription was shown to be recognised by both SSX2A and SSX2B primers. (**G**) SSX2B transcription was recognised by SSX2B and SSX4 primers. (**H**) SSX3 transcription was shown to be recognised by SSX2B and SSX3 primers. (**I**) SSX4 transcription was indicated by SSX2B and SSX4 primers. GAPDH was used as the reference gene. Data representative of *n* = 3 experimental repeats, error bars ± SEM.

**Figure 3:**
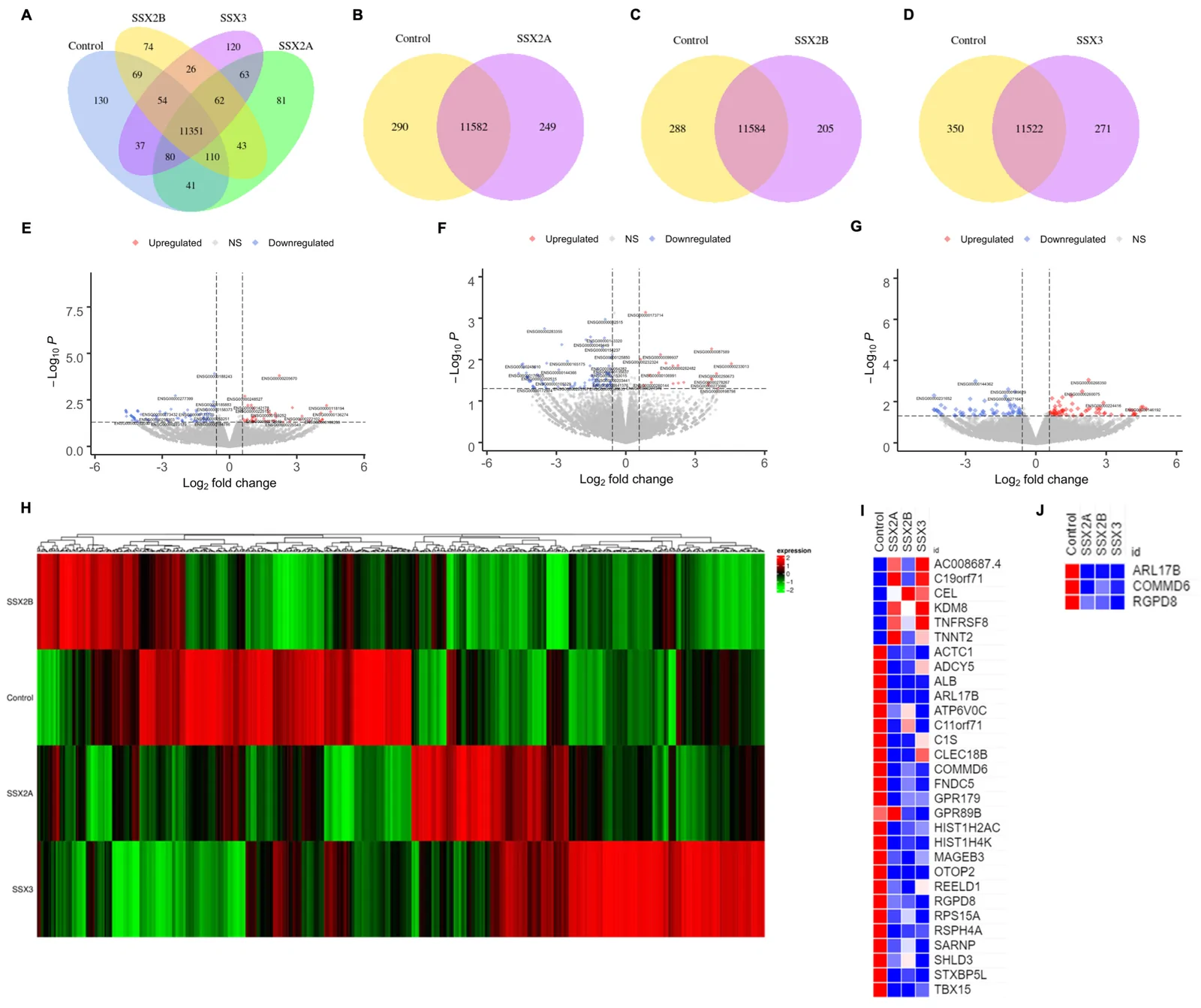
Global transcriptome profiling of OC following ectopic expression of SSX2A, SSX2B and SSX3 indicates common and unique changes. (**A**) Different and common genes were found when comparing each of the OVCAR-3-SSX variants compared to each other and the control. (**B**) The number of unique and common genes transcribed when comparing OVCAR-3-SSX2A, (**C**) OVCAR-3-SSX2B, (**D**) OVCAR-3-SSX3 versus OVCAR-3-VC. Differential gene expression when comparing OVCAR-3 cells transfected with (**E**) SSX2A, (**F**) SSX2B or (**G**) SSX3 versus OVCAR-3-VC cells. A log_2_FC cutoff value of 0.58 (x axis, dashed line) and a non-adjusted *p* ≤ 0.05 was used to highlight significantly upregulated or downregulated genes in the Volcano plots. Key transcripts are labelled with Ensembl gene IDs. (**H**) Hierarchical clustering of the DEGs following overexpression of SSX2A, SSX2B or SSX3 in OVCAR-3 versus VC transfected cells showed there were more commonly identified genes between (**I**) two or (**J**) three OVCAR-3-SSX groups. Upregulated and downregulated genes are highlighted in red and blue respectively, with intensity of colour corresponding to range of row values. Heatmaps were produced using FPKM values. *n* = 3 samples were analysed per group.

**Figure 4:**
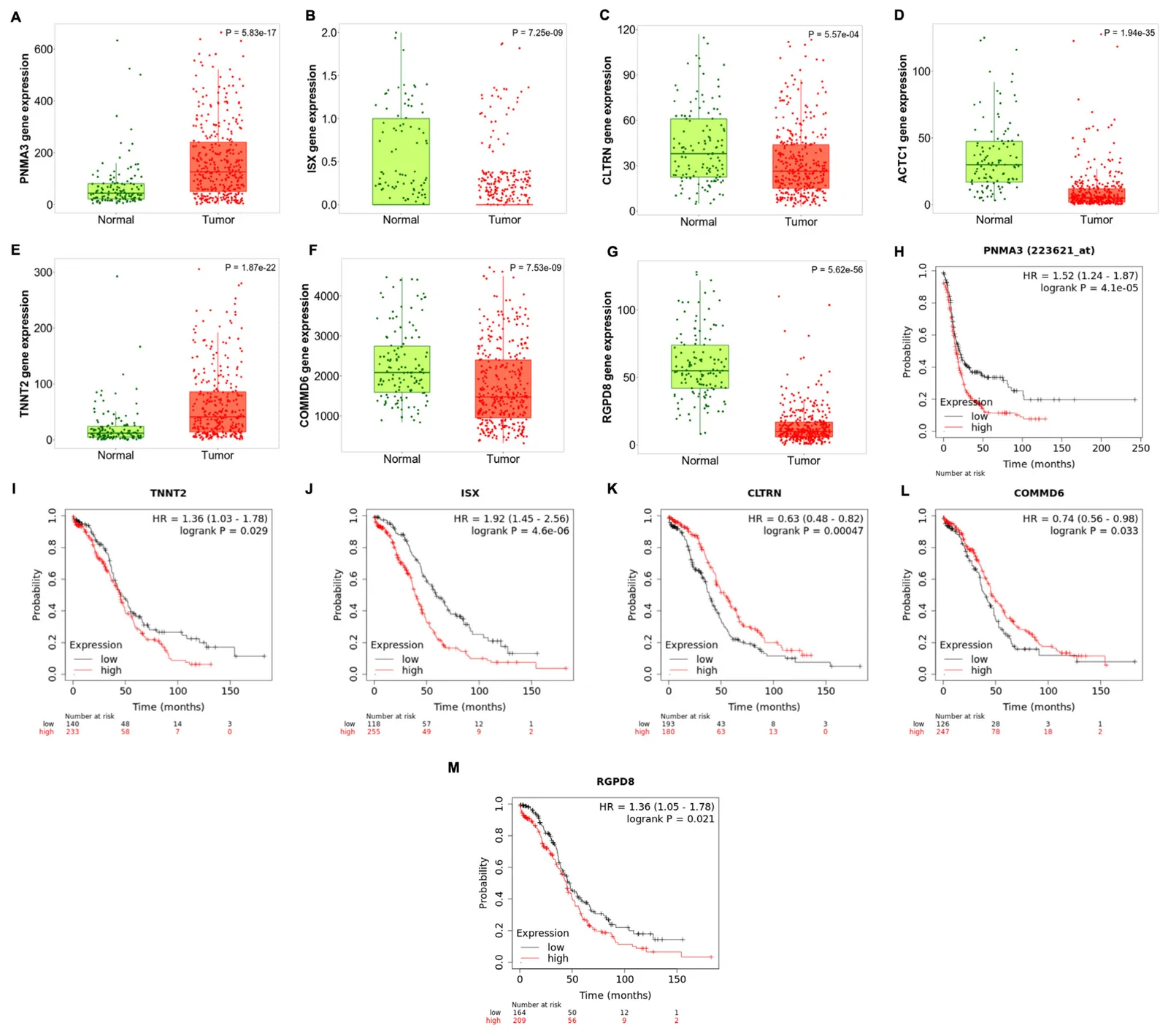
Expressions of DEGs identified via RNA-Seq following ectopic SSX expression have clinically relevant transcriptional changes. Comparison of the expression of (**A**) PNMA3, (**B**) ISX, (**C**) CLTRN, (**D**) ACTC1, (**E**) TNNT2, (**F**) COMMD6 and (**G**) RGPD8 in healthy donor ovarian tissue (*n* = 133) versus OC (*n* = 374) samples. The Mann–Whitney U test values are indicated in the top right of each graph. OS of OC patients (*n* = 374) based on high (red line) or low (black line) mRNA expression of (**H**) PNMA3, (**I**) TNNT2, (**J**) ISX, (**K**) CLTRN (**L**) COMMD6 or (**M**) RGPD8. Cohorts were assigned according to the best cutoff value. Logrank P = *p* value, HR = hazard ratio.

**Figure 5:**
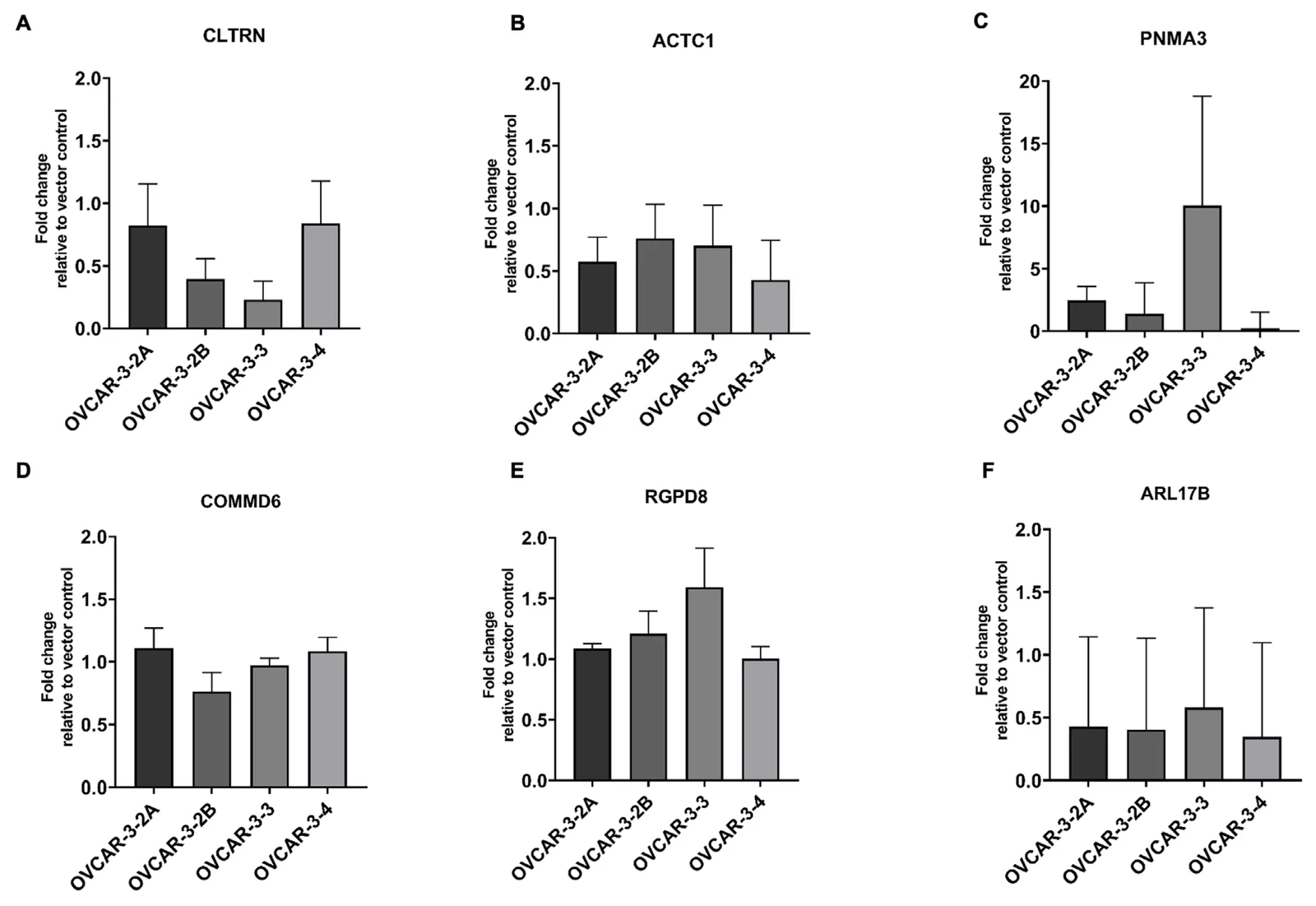
Validation of SSX2A, SSX2B, SSX3 and SSX4 DEGs. The OVCAR-3 cell line was transiently transfected with SSX2A, SSX2B, SSX3, SSX4 or a vector alone, and samples were collected 18 h post-transfection. Expression levels of genes identified as commonly up or downregulated (**A**) CLTRN, (**B**) ACTC1, (**C**) PNMA3, (**D**) COMMD6, (**E**) RGPD8 and (**F**) ARL17B were assessed by qPCR to validate RNA-Seq findings. The fold change was calculated relative to the VC transfected sample using the 2^−^^ΔΔCT^ method. GAPDH was used as the reference gene. Data representative of *n* = 3 experimental repeats, error bars ± SEM.

**Figure 6:**
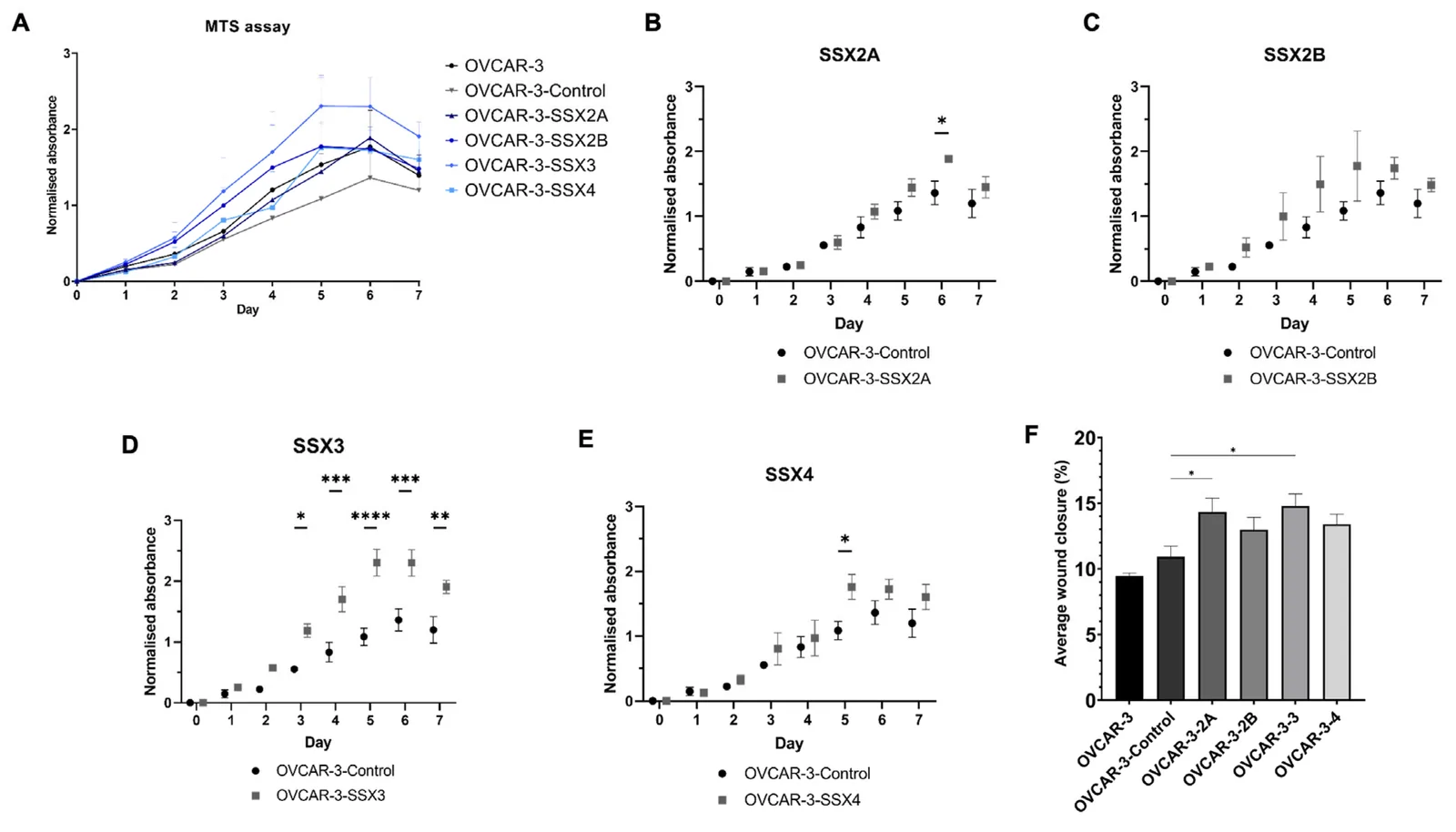
SSX family members increase the proliferation and migration of OC. (**A**) MTS proliferation assay showing the results from all variants versus control (OVCAR-3-VC) overlayed and OVCAR-3 cells transfected with (**B**) SSX2A, (**C**) SSX2B, (**D**) SSX3 or (**E**) SSX4 compared to the VC cells individually. A two-way ANOVA was performed with Šidák correction. (**F**) The migration of transfected cells over 24 h were assessed using a wound healing assay. A one-way ANOVA was performed with Dunnett correction. * = *p* < 0.05, ** = *p* < 0.01, *** = *p* < 0.001 and **** = *p* < 0.0001. Data is representative of *n* = 3 experimental repeats, error bars ± SEM.

**Figure 7:**
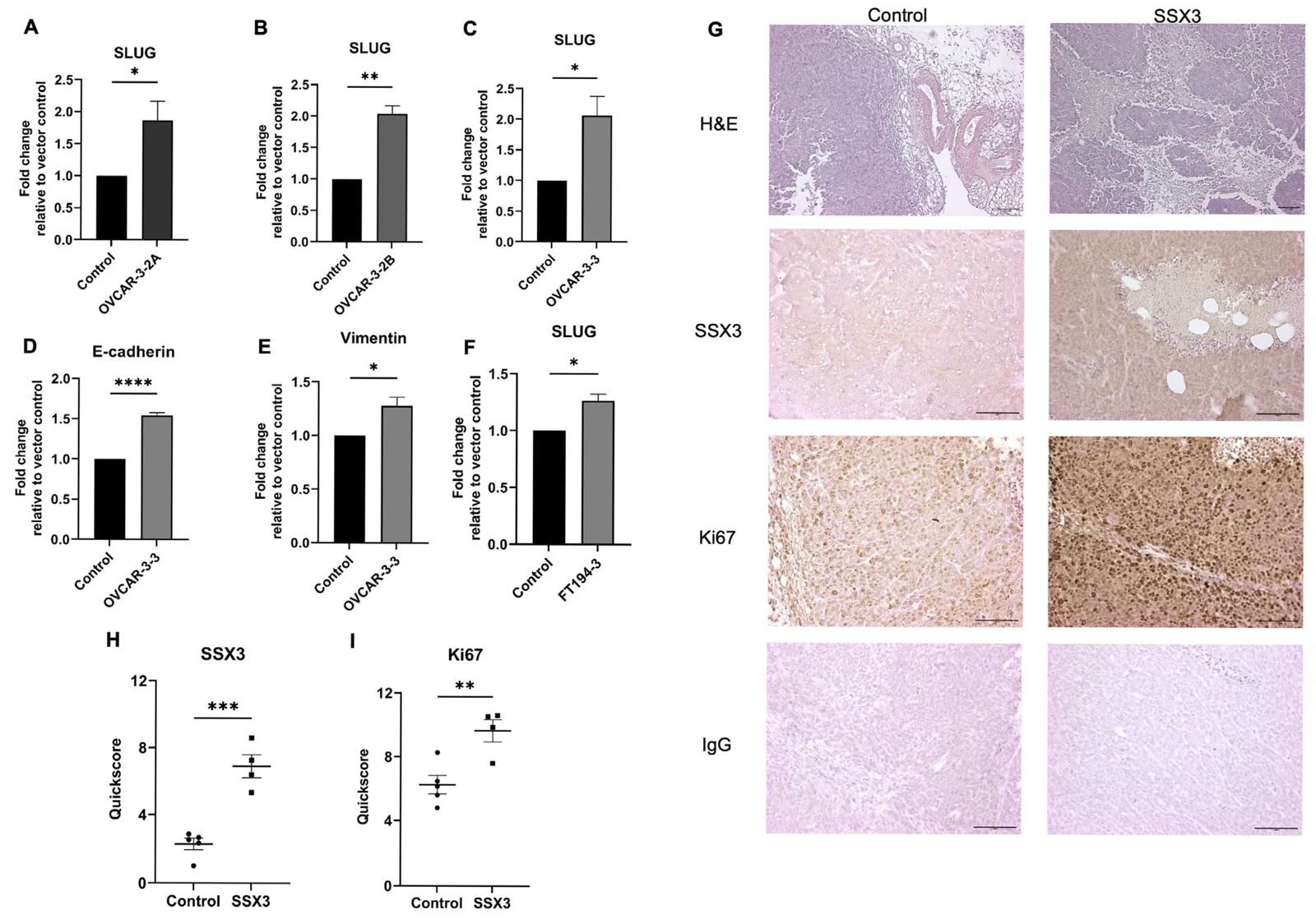
SSX expression resulted in increased expression of EMT-associated genes and for SSX3 the Ki67 marker of proliferation in vivo. Transcript levels for SLUG were significantly increased in OVCAR-3 cells transiently transfected with (**A**) SSX2A, (**B**) SSX2B and (**C**) SSX3 in comparison to the vector ‘control’. OVCAR-3 cells transfected with SSX3 also had significantly increased levels of (**D**) E-cadherin and (**E**) Vimentin. (**F**) Fallopian tube cells, FT194, transfected with SSX3 showed increased levels of SLUG. The fold change was calculated relative to the VC using the 2^−ΔΔCT^ method. GAPDH was used as the endogenous reference gene. Immunohistochemistry (IHC) was performed on OVCAR-3-control (*n* = 5) and OVCAR-3-SSX3 (*n* = 4) xenografts. (**G**) Representative H&E (top row) and immunolabelling of SSX3 (second row), Ki67 (third row) and the negative IgG control (bottom row). Images were taken using a Nikon Eclipse E400 microscope, H&E at 10× and IHC at 20 × magnification. OVCAR-3-SSX3 xenografts had increased expressions of (**H**) SSX3 and (**I**) Ki67. Samples were scored with a modified Quickscore method. Scale bar = 100 μm. * = *p* < 0.05, ** = *p* < 0.01, *** = *p* < 0.001 and **** = *p* < 0.0001. Data representative of *n* = 3 experimental repeats, error bars ± SEM.

## Data Availability

The original contributions presented in this study are included in the article/Supplementary Material. Further inquiries can be directed to the corresponding authors.

## Supplementary Materials

The following supporting information can be downloaded at: https://www.mdpi.com/article/10.3390/ijms27198608/s1.

## Abbreviations

The following abbreviations are used in this manuscript:

ACTC1	Actin Alpha Cardiac Muscle 1
ARL17B	ADP-ribosylation factor-like 17B
ATCC	American Type Culture Collection
CLTRN	Collectrin
COMMD6	Copper metabolism gene MURR1 domain containing 6
CTA	Cancer-testis antigen
DAVID	Database for Annotation, Visualization and Integrated Discovery
DEG	Differentially expressed genes
EMT	Epithelial-mesenchymal transition
EOC	Epithelial ovarian cancer
FPKM	Fragments per kilobase of transcript sequence per million base pairs sequenced
GO	Gene ontology
H&E	Haematoxylin and eosin
HGSOC	High grade serous ovarian cancer
IHC	Immunohistochemistry
IPA	Ingenuity^®^ Pathway Analysis
ISX	Intestine-specific homeobox
OS	Overall survival
PCA	Principal component analysis
PCR	Polymerase chain reaction
PNMA3	Paraneoplastic antigen MA3
qRT	Quantitative Real-Time
RGPD8	RANBP2-like and GRIP domain containing 8
RNA	Ribonucleic acid
RPMI	Roswell Park Memorial Institute
RNA-Seq	RNA sequencing
SSX	Synovial sarcoma, X breakpoint
TNNT2	Troponin T
VC	Vector control

## References

[1] Prat J. (2012). New insights into ovarian cancer pathology. Ann. Oncol..

[2] Lisio M.A., Fu L., Goyeneche A., Gao Z.H., Telleria C. (2019). High-Grade Serous Ovarian Cancer: Basic Sciences, Clinical and Therapeutic Standpoints. Int. J. Mol. Sci..

[3] Vaughan M.H., Modesitt S.C., Mo Y., Trowbridge E.R. (2015). Serous tubal intraepithelial carcinoma: An incidental finding at the time of prophylactic bilateral salpingo-oophorectomy. Case Rep. Obstet. Gynecol..

[4] Nowak M., Janas Ł., Stachowiak G., Stetkiewicz T., Wilczyński J.R. (2015). Current clinical application of serum biomarkers to detect ovarian cancer. Przegląd Menopauzalny.

[5] Karimi-Zarchi M., Dehshiri-Zadeh N., Sekhavat L., Nosouhi F. (2016). Correlation of CA-125 serum level and clinico-pathological characteristic of patients with endometriosis. Int. J. Reprod. Biomed..

[6] Johnson C.C., Kessel B., Riley T.L., Ragard L.R., Williams C.R., Xu J.L., Buys S.S. (2008). The epidemiology of CA-125 in women without evidence of ovarian cancer in the Prostate, Lung, Colorectal and Ovarian Cancer (PLCO) Screening Trial. Gynecol. Oncol..

[7] Mirandola L., Cannon M.J., Cobos E., Bernardini G., Jenkins M.R., Kast W.M., Chiriva-Internati M. (2011). Cancer testis antigens: Novel biomarkers and targetable proteins for ovarian cancer. Int. Rev. Immunol..

[8] Gordeeva O. (2018). Cancer-testis antigens: Unique cancer stem cell biomarkers and targets for cancer therapy. Semin. Cancer Biol..

[9] Clark J., Rocques P.J., Crew A.J., Gill S., Shipley J., Chan A.M., Gusterson B.A., Cooper C.S. (1994). Identification of novel genes, SYT and SSX, involved in the t(X;18)(p11.2;q11.2) translocation found in human synovial sarcoma. Nat. Genet..

[10] Güre A.O., Wei I.J., Old L.J., Chen Y.-T. (2002). The SSX gene family: Characterization of 9 complete genes. Int. J. Cancer.

[11] Türeci O., Sahin U., Schobert I., Koslowski M., Scmitt H., Schild H.J., Stenner F., Seitz G., Rammensee H.G., Pfreundschuh M. (1996). The SSX-2 gene, which is involved in the t(X;18) translocation of synovial sarcomas, codes for the human tumor antigen HOM-MEL-40. Cancer Res..

[12] Tureci O., Chen Y.T., Sahin U., Gure A.O., Zwick C., Villena C., Tsang S., Seitz G., Old L.J., Pfreundschuh M. (1998). Expression of SSX genes in human tumors. Int. J. Cancer J. Int. Cancer.

[13] Valmori D., Qian F., Ayyoub M., Renner C., Merlo A., Gnjatic S., Stockert E., Driscoll D., Lele S., Old L.J. (2006). Expression of synovial sarcoma X (SSX) antigens in epithelial ovarian cancer and identification of SSX-4 epitopes recognized by CD4+ T cells. Clin. Cancer Res. Off. J. Am. Assoc. Cancer Res..

[14] Grayson K. (2023). Investigating Synovial Sarcoma, X Breakpoint Proteins in Ovarian Cancer.

[15] Fearn A., Dolińska W., Grayson K., Khan G.N., Pulford K., Wilkinson H., Guinn B.A. SSX Family Members Play Different Roles in the Early Development of Ovarian Cancer.

[16] Vogel C., Marcotte E.M. (2012). Insights into the regulation of protein abundance from proteomic and transcriptomic analyses. Nat. Rev. Genet..

[17] Traynor S., Jakobsen N.D., Ebbesen M.F., Bennedsen S.N., Johansen S., Ebstrup M.L., Pedersen C.B., Ditzel H.J., Brewer J.R., Gjerstorff M.F. (2022). SSX2 promotes the formation of a novel type of intranuclear lamin bodies. Int. J. Biochem. Cell Biol..

[18] Alrubie T.M., Alamri A.M., Almutairi B.O., Alrefaei A.F., Arafah M.M., Alanazi M., Semlali A., Almutairi M.H. (2023). Higher Expression Levels of SSX1 and SSX2 in Patients with Colon Cancer: Regulated In Vitro by the Inhibition of Methylation and Histone Deacetylation. Medicina.

[19] Chen L., Zhou W.B., Zhao Y., Liu X.A., Ding Q., Zha X.M., Wang S. (2012). Cancer/testis antigen SSX2 enhances invasiveness in MCF-7 cells by repressing ERα signaling. Int. J. Oncol..

[20] Brückmann N.H., Bennedsen S.N., Duijf P.H.G., Terp M.G., Thomassen M., Larsen M., Pedersen C.B., Kruse T., Alcaraz N., Ditzel H.J. (2019). A functional genetic screen identifies the Mediator complex as essential for SSX2-induced senescence. Cell Death Dis..

[21] Greve K.B.V., Lindgreen J.N., Terp M.G., Pedersen C.B., Schmidt S., Mollenhauer J., Kristensen S.B., Andersen R.S., Relster M.M., Ditzel H.J. (2015). Ectopic expression of cancer/testis antigen SSX2 induces DNA damage and promotes genomic instability. Mol. Oncol..

[22] Bloom J.E., McNeel D.G. (2016). SSX2 regulates focal adhesion but does not drive the epithelial to mesenchymal transition in prostate cancer. Oncotarget.

[23] Cronwright G., Le Blanc K., Götherström C., Darcy P., Ehnman M., Brodin B. (2005). Cancer/testis antigen expression in human mesenchymal stem cells: Down-regulation of SSX impairs cell migration and matrix metalloproteinase 2 expression. Cancer Res..

[24] Bradbury A., O’Donnell R., Drew Y., Curtin N., Saha S.S. (2020). Characterisation of Ovarian Cancer Cell Line NIH-OVCAR3 and Implications of Genomic, Transcriptomic, Proteomic and Functional DNA Damage Response Biomarkers for Therapeutic Targeting. Cancers.

[25] Bricard G., Bouzourene H., Martinet O., Rimoldi D., Halkic N., Gillet M., Chaubert P., Macdonald H.R., Romero P., Cerottini J.C. (2005). Naturally acquired MAGE-A10- and SSX-2-specific CD8+ T cell responses in patients with hepatocellular carcinoma. J. Immunol..

[26] Gehring A.J., Ho Z.Z., Tan A.T., Aung M.O., Lee K.H., Tan K.C., Lim S.G., Bertoletti A. (2009). Profile of tumor antigen-specific CD8 T cells in patients with hepatitis B virus-related hepatocellular carcinoma. Gastroenterology.

[27] Li P., Lin Y., Zhang Y., Zhu Z., Huo K. (2013). SSX2IP promotes metastasis and chemotherapeutic resistance of hepatocellular carcinoma. J. Transl. Med..

[28] Lv X., Kong J., Chen W.-D., Wang Y.-D. (2017). The Role of the Apelin/APJ System in the Regulation of Liver Disease. Front. Pharmacol..

[29] Sherman B.T., Hao M., Qiu J., Jiao X., Baseler M.W., Lane H.C., Imamichi T., Chang W. (2022). DAVID: A web server for functional enrichment analysis and functional annotation of gene lists (2021 update). Nucleic Acids Res..

[30] Gjerstorff M.F., Relster M.M., Greve K.B., Moeller J.B., Elias D., Lindgreen J.N., Schmidt S., Mollenhauer J., Voldborg B., Pedersen C.B. (2014). SSX2 is a novel DNA-binding protein that antagonizes polycomb group body formation and gene repression. Nucleic Acids Res..

[31] Traynor S., Møllegaard N.E., Jørgensen M.G., Brückmann N.H., Pedersen C.B., Terp M.G., Johansen S., Dejardin J., Ditzel H.J., Gjerstorff M.F. (2019). Remodeling and destabilization of chromosome 1 pericentromeric heterochromatin by SSX proteins. Nucleic Acids Res..

[32] Khan G. (2016). Characterisation of the Expression of Tumour Antigens and Biomarkers in Myeloid Leukaemia and Ovarian Cancer.

[33] Greve K.B., Pøhl M., Olsen K.E., Nielsen O., Ditzel H.J., Gjerstorff M.F. (2014). SSX2-4 expression in early-stage non-small cell lung cancer. Tissue Antigens.

[34] Frank S.A. (2020). Dynamics of Cancer: Incidence, Inheritance, and Evolution.

[35] Kuhlman B., Bradley P. (2019). Advances in protein structure prediction and design. Nat. Rev. Mol. Cell Biol..

[36] Lang F., Schrörs B., Löwer M., Türeci Ö., Sahin U. (2022). Identification of neoantigens for individualized therapeutic cancer vaccines. Nat. Rev. Drug Discov..

[37] Day C.A., Hinchcliffe E.H., Robinson J.P. (2022). H3K27me3 in Diffuse Midline Glioma and Epithelial Ovarian Cancer: Opposing Epigenetic Changes Leading to the Same Poor Outcomes. Cells.

[38] Monteith G.R., Prevarskaya N., Roberts-Thomson S.J. (2017). The calcium-cancer signalling nexus. Nat. Rev. Cancer.

[39] Wu S., Lv Z., Wang Y., Sun L., Jiang Z., Xu C., Zhao J., Sun X., Li X., Hu L. (2013). Increased Expression of Pregnancy Up-Regulated Non-Ubiquitous Calmodulin Kinase Is Associated with Poor Prognosis in Clear Cell Renal Cell Carcinoma. PLoS ONE.

[40] Gardner H.P., Ha S.I., Reynolds C., Chodosh L.A. (2000). The caM kinase, Pnck, is spatially and temporally regulated during murine mammary gland development and may identify an epithelial cell subtype involved in breast cancer. Cancer Res..

[41] Kang X., Cui C., Wang C., Wu G., Chen H., Lu Z., Chen X., Wang L., Huang J., Geng H. (2018). CAMKs support development of acute myeloid leukemia. J. Hematol. Oncol..

[42] Tsuyoshi H., Orisaka M., Fujita Y., Asare-Werehene M., Tsang B.K., Yoshida Y. (2020). Prognostic impact of Dynamin related protein 1 (Drp1) in epithelial ovarian cancer. BMC Cancer.

[43] Bartha Á., Győrffy B. (2021). TNMplot.com: A Web Tool for the Comparison of Gene Expression in Normal, Tumor and Metastatic Tissues. Int. J. Mol. Sci..

[44] Szender J.B., Papanicolau-Sengos A., Eng K.H., Miliotto A.J., Lugade A.A., Gnjatic S., Matsuzaki J., Morrison C.D., Odunsi K. (2017). NY-ESO-1 expression predicts an aggressive phenotype of ovarian cancer. Gynecol. Oncol..

[45] Zhang S., Zhou X., Yu H., Yu Y. (2010). Expression of tumor-specific antigen MAGE, GAGE and BAGE in ovarian cancer tissues and cell lines. BMC Cancer.

[46] D’Arcy P., Maruwge W., Wolahan B., Ma L., Brodin B. (2014). Oncogenic functions of the cancer-testis antigen SSX on the proliferation, survival, and signaling pathways of cancer cells. PLoS ONE.

[47] Steinbichler T.B., Dudas J., Ingruber J., Glueckert R., Sprung S., Fleischer F., Cidlinsky N., Dejaco D., Kofler B., Giotakis A.I. (2020). Slug Is A Surrogate Marker of Epithelial to Mesenchymal Transition (EMT) in Head and Neck Cancer. J. Clin. Med..

[48] Zheng M., Jiang Y.P., Chen W., Li K.D., Liu X., Gao S.Y., Feng H., Wang S.S., Jiang J., Ma X.R. (2015). Snail and Slug collaborate on EMT and tumor metastasis through miR-101-mediated EZH2 axis in oral tongue squamous cell carcinoma. Oncotarget.

[49] Gu A., Jie Y., Yao Q., Zhang Y., Mingyan E. (2017). Slug Is Associated With Tumor Metastasis and Angiogenesis in Ovarian Cancer. Reprod. Sci..

[50] Baranwal S., Alahari S.K. (2009). Molecular mechanisms controlling E-cadherin expression in breast cancer. Biochem. Biophys. Res. Commun..

[51] Mendez M.G., Kojima S., Goldman R.D. (2010). Vimentin induces changes in cell shape, motility, and adhesion during the epithelial to mesenchymal transition. FASEB J..

[52] Rosso M., Majem B., Devis L., Lapyckyj L., Besso M.J., Llauradó M., Abascal M.F., Matos M.L., Lanau L., Castellví J. (2017). E-cadherin: A determinant molecule associated with ovarian cancer progression, dissemination and aggressiveness. PLoS ONE.

[53] Blighe K., Rana S., Lewis M. (2021). EnhancedVolcano: Publication-Ready Volcano Plots with Enhanced Colouring and Labelling [Internet].

[54] Bartha A., Gyorffy B. (2026). TNMplot: An enhanced platform for pharmacological target identification through cross-stage and pan-cancer gene expression analysis. Br. J. Pharmacol..

[55] Gyorffy B. (2023). Discovery and ranking of the most robust prognostic biomarkers in serous ovarian cancer. Geroscience.

[56] Livak K.J., Schmittgen T.D. (2001). Analysis of relative gene expression data using real-time quantitative PCR and the 2(-Delta Delta C(T)) Method. Methods.

[57] Jonkman J.E., Cathcart J.A., Xu F., Bartolini M.E., Amon J.E., Stevens K.M., Colarusso P. (2014). An introduction to the wound healing assay using live-cell microscopy. Cell Adhes. Migr..

[58] Suarez-Arnedo A., Figueroa F.T., Clavijo C., Arbeláez P., Cruz J.C., Muñoz-Camargo C. (2020). An image J plugin for the high throughput image analysis of in vitro scratch wound healing assays. PLoS ONE.

[59] Schneider C.A., Rasband W.S., Eliceiri K.W. (2012). NIH Image to ImageJ: 25 years of image analysis. Nat. Methods.

[60] Workman P., Aboagye E.O., Balkwill F., Balmain A., Bruder G., Chaplin D.J., Double J.A., Everitt J., Farningham D.A., Glennie M.J. (2010). Guidelines for the welfare and use of animals in cancer research. Br. J. Cancer.

[61] Detre S., Jotti G.S., Dowsett M. (1995). A “quickscore” method for immunohistochemical semiquantitation: Validation for oestrogen receptor in breast carcinomas. J. Clin. Pathol..

