# Supplementary material for: Distinct and Convergent Roles of the Synovial Sarcoma X Breakpoint Family of Cancer-Testis Antigens in Ovarian Cancer
Source: Int J Mol Sci. 2026 Sep 26;27(19):8608. doi: 10.3390/ijms27198608 (PMC13665215; doi:10.3390/ijms27198608)
Supplement: Supplementary file 1 [file ijms-27-08608-s001.zip › Supplemental Figures.pdf]

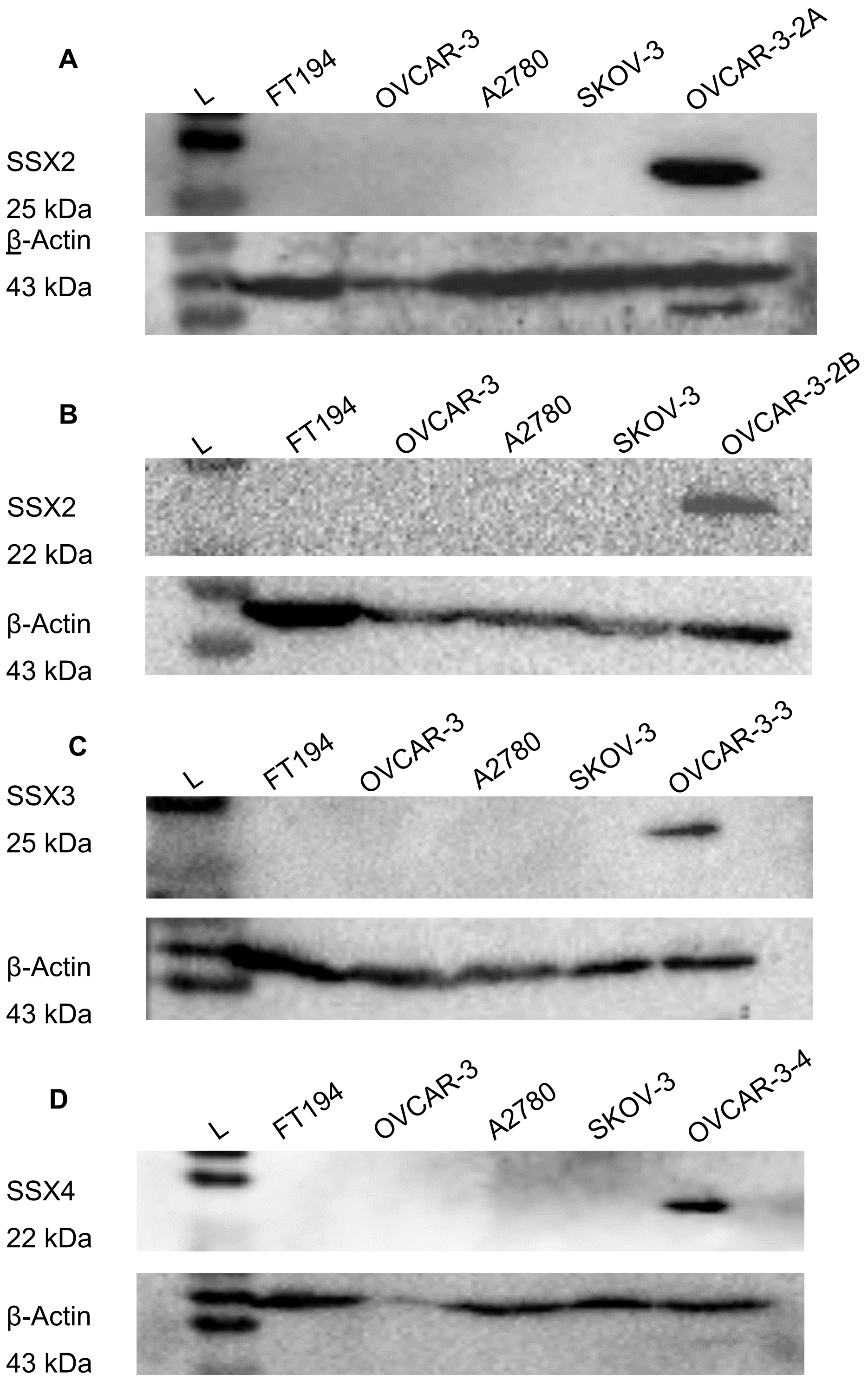

Figure S1 SSX expression cannot be demonstrated by western blot in OC cell lines

Representative western blots of (A) SSX2A, (B) SSX2B, (C) SSX3 and (D) SSX4 indicate no SSX protein expression except in the respective OVCAR-3 transfected cell line denoted by OVCAR-3-2A, OVCAR-3-2B, OVCAR-3-3 and OVCAR-3-4 respectively. β-actin was used as a loading control, images shown are representative of n = 3. L = Biorad precision plus protein dual colour standards.

SSX2A	MNGDDAFARR	PTVGAQIPEK	IQKAFDDIAK	YFSKEEWEKM	KASEKIFYVY
SSX2B	MNGDDAFARR	PTVGAQIPEK	IQKAFDDIAK	YFSKEEWEKM	KASEKIFYVY
SSX3	MNGDDTFARR	PTVGAQIPEK	IQKAFDDIAK	YFSKEEWEKM	KVSEKIVYVY
SSX2A	MKRKYEAMTK	LGFKATLPPF	MCNKRAEDFQ	GNDLDNDPNR	GNQVERPQMT
SSX2B	MKRKYEAMTK	LGFKATLPPF	MCNKRAEDFQ	GNDLDNDPNR	GNQVERPQMT
SSX3	MKRKYEAMTK	LGFKAILPSF	MRNKRVTDFQ	GNDFDNDPNR	GNQVQRPQMT
SSX2A	FGRLQGISPK	IMPKKPAEEG	NDSEEVPEAS	GPQNDGKELC	PPGKPTTSEK
SSX2B	FGRLQGISPK	IMPKKPAEEG	NDSEEVPEAS	GPQNDGKELC	PPGKPTTSEK
SSX3	FGRLQGIFPK	IMPKKPAEEG	NVSKEVPEAS	GPQNDGKQLC	PPGKPTTSEK
SSX2A	IHERSGNREA	QEKEERRGTA	HRWSSQNTHN	IGRFSLSTSM	GAVHGTPKTI
SSX2B	IHERSGPKRG	EHAWTHRLRE	RKQLVIYEEI	SDPEEDDE	
SSX3	INMISGPKRG	EHAWTHRLRE	RKQLVIYEEI	SDPEEDDE	
SSX2A	THNRDPKGGN	MPGPTDCVRE	NSW		

Figure S2 Epitope mapping of SSX2 4D10 antibody

Epitope mapping of SSX2 ‘4D10’ antibody correlating to on amino acid sequences of SSX2A, SSX2B and SSX3. The amino acids corresponding to those present in the antibody are highlighted in red.

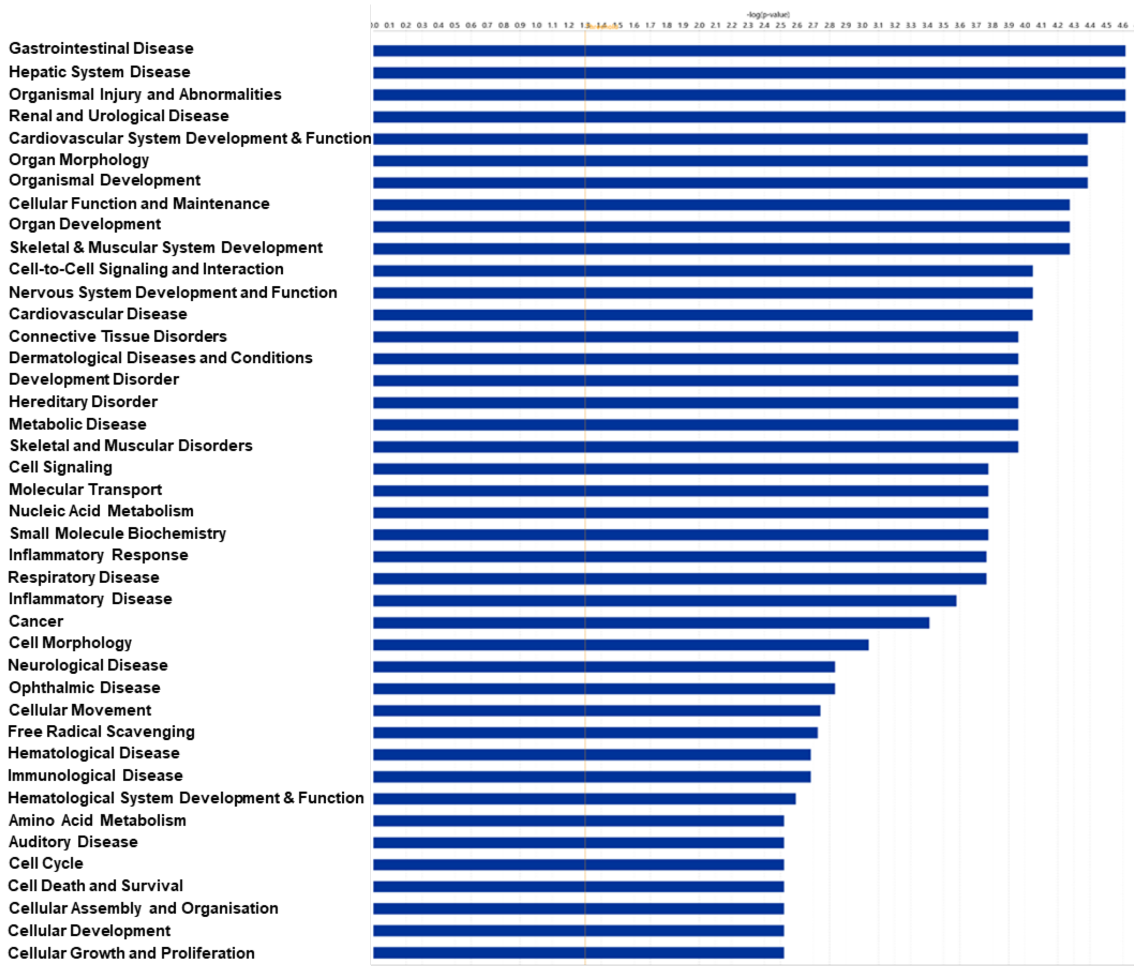

Figure S3 Biological functions and disease categories altered by SSX2A overexpression ranked by p value

RNA-Seq was performed on OVCAR-3 cells overexpressing of SSX2A or the vector control plasmid. Genes which were shown to have ± 1.5 fold change in comparison to the control and a *p* value of ≤0.05 were shown to be involved in biological functions and diseases using IPA software and ranked according to p value to highlight the most statistically significant. Yellow bar represents a significance threshold of $p = 0.05$ and the total bar height indicates the average p value of all functions within the categories.

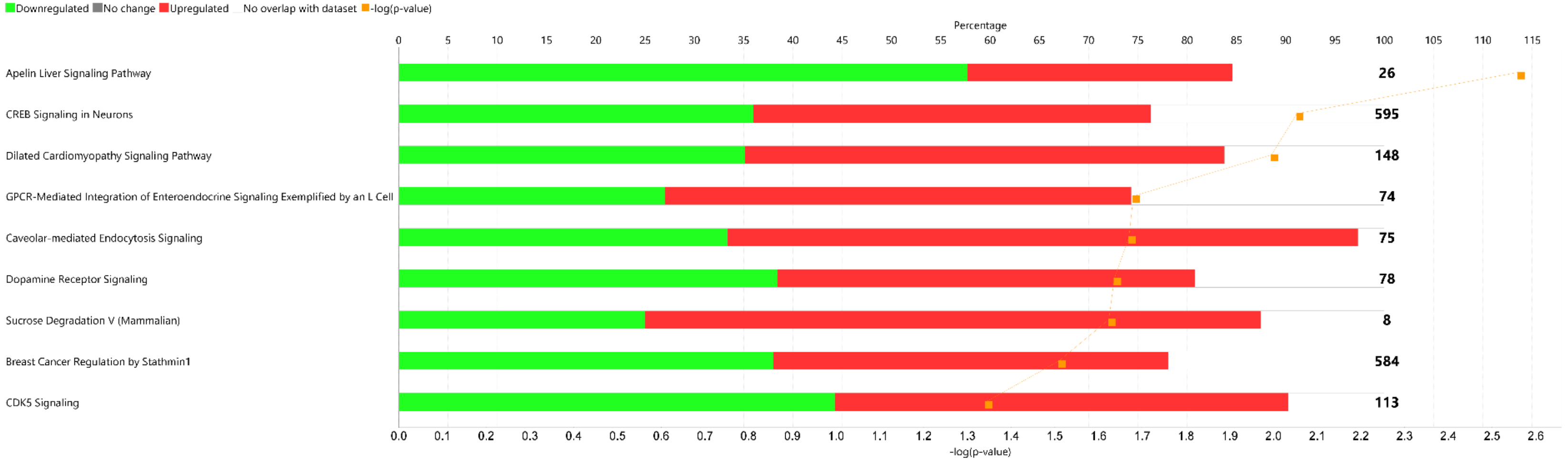

© 2000-2022 QIAGEN. All rights reserved.

Figure S4 Signalling pathways altered by SSX2A overexpression
OVCAR-3 cells were transfected to overexpress SSX2A or a vector control plasmid. Samples were analysed by RNA-Seq to identify all transcripts. DE genes between SSX2A and the control group were identified as ± 1.5 fold change and a *p* value of ≤0.05. IPA software was used to analyse the canonical signalling and metabolic pathways affected by the DE genes. Orange bar represents -log(p-value), green bar indicates the percentage of downregulated genes, red bar indicates upregulated genes, white bar indicates genes with no involvement. The total number of genes involved in each pathway is highlighted in bold at the end of the bar.

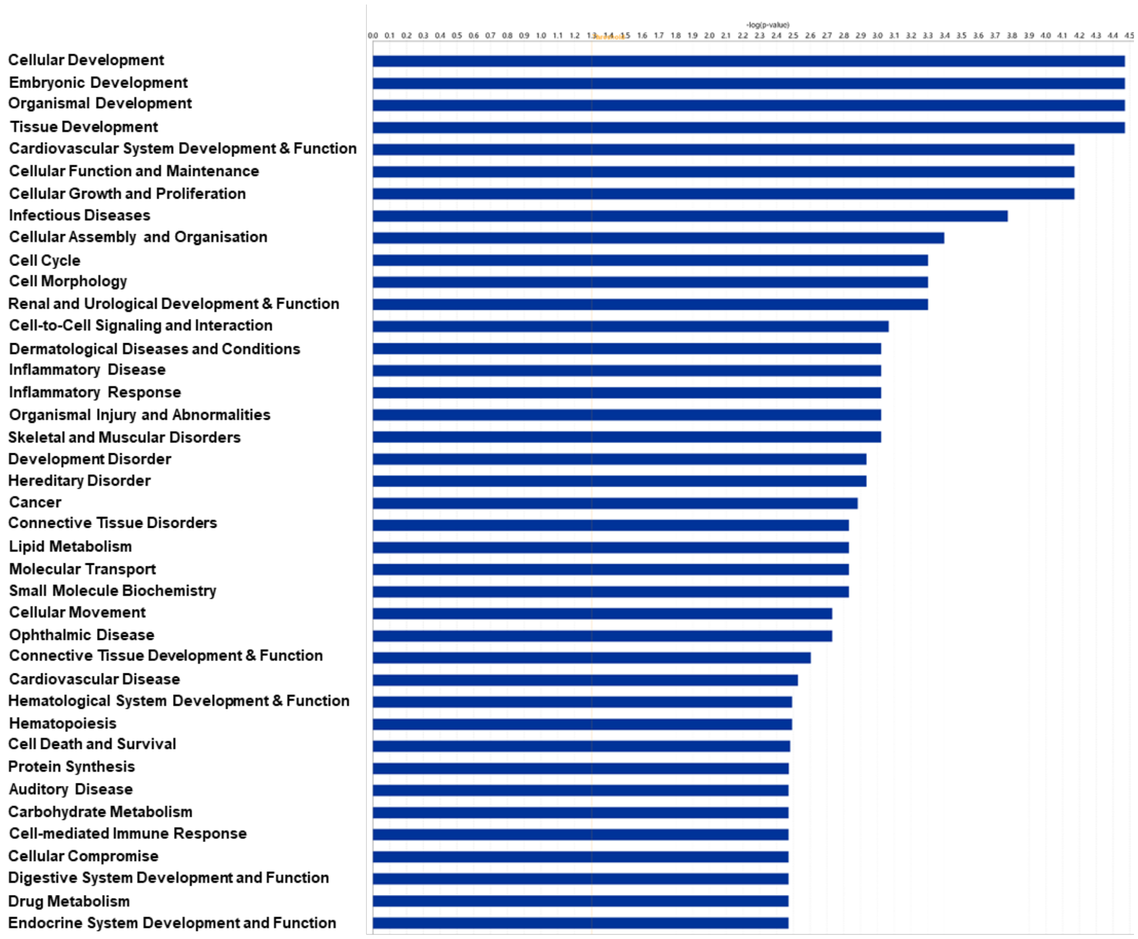

Figure S5 Biological functions and disease categories altered by SSX2B overexpression ranked by *p* value

RNA-Seq was performed on OVCAR-3 cells overexpressing of SSX2B or the vector control plasmid. Genes which were shown to have ± 1.5 fold change in comparison to the control and a *p* value = <0.05 were shown to be involved in biological functions and diseases using IPA software and ordered by *p* value. Yellow bar represents a significance threshold of *p* = 0.05 and the total bar height indicates the average *p* value of all functions within the categories.

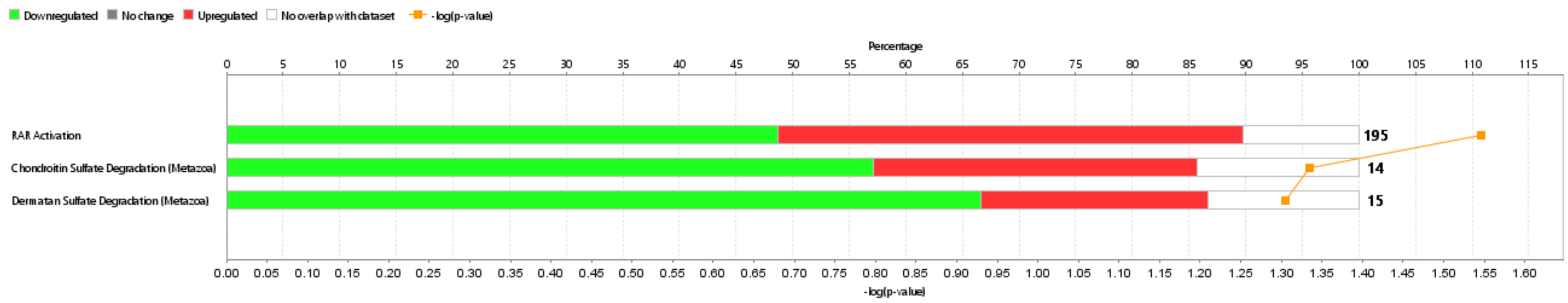

Figure S6 Signalling pathways altered by SSX2B overexpression

OVCAR-3 cells were transfected to overexpress SSX2B or a vector control plasmid. Samples were analysed by RNA-Seq to identify all transcripts, where the DE of genes when comparing SSX2B and the control group were identified using >± 1.5 fold change and a *p* value cut-off of ≤0.05. IPA software was used to analyse the canonical signalling and metabolic pathways used by the DE genes. Orange bar represents -log(p-value), with *p* values between 2.84×10^{-2} – 4.96×10^{-2}, green bar indicates the percentage of downregulated genes and the red bar indicates the percentage of upregulated genes. The total number of genes involved in each pathway is highlighted in bold at the end of the bar.

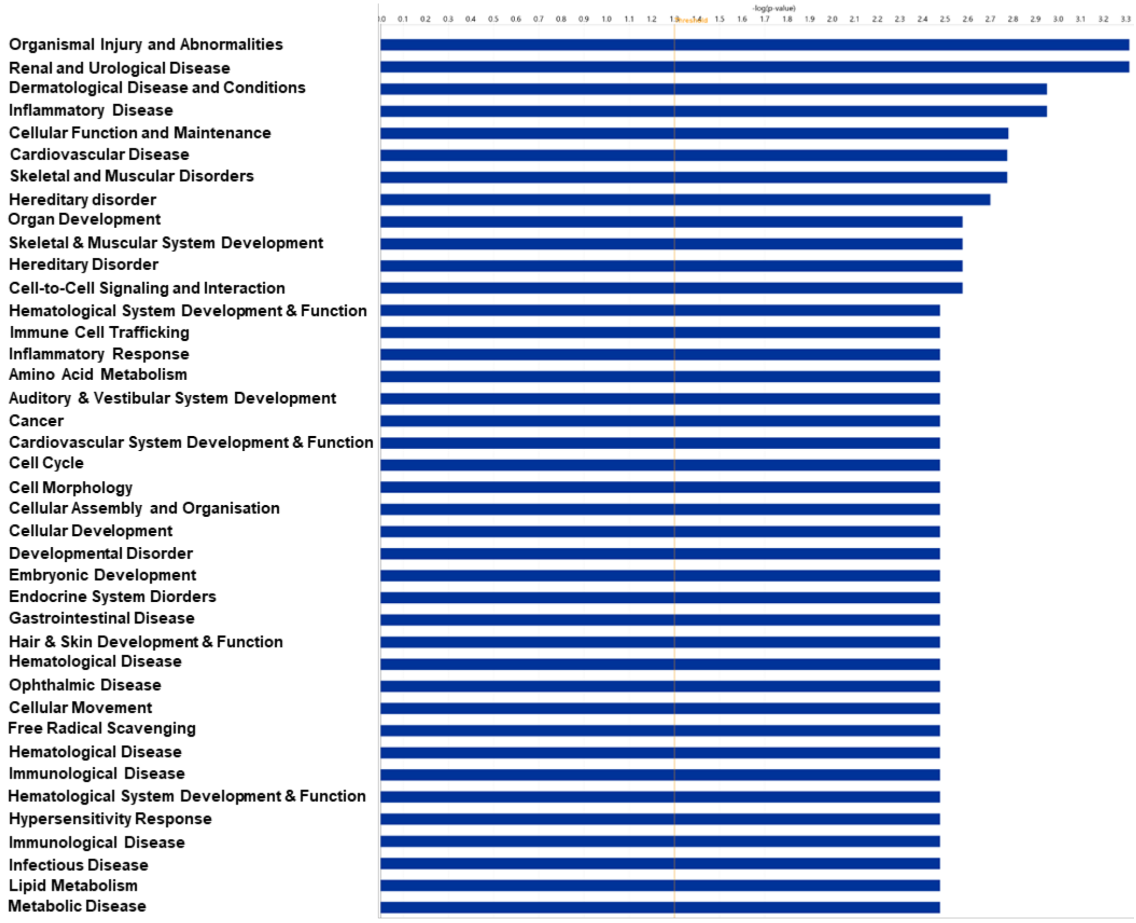

Figure S7 Biological functions and disease categories altered by SSX3 overexpression ranked by *p* value

RNA-Seq was performed on OVCAR-3 cells overexpressing SSX3 and DE genes identified when compared to those expressed in the OVCAR-3 transfected with vector control plasmid alone. DE genes, that were shown to have ± 1.5 fold change in comparison to the control and a *p* value = ≤0.05, were investigated for their involvement in biological functions and diseases using IPA software and ranked by *p* value. The yellow bar represents a significance threshold of *p* = 0.05 and the total bar height indicates the average *p* value of all functions within the categories.

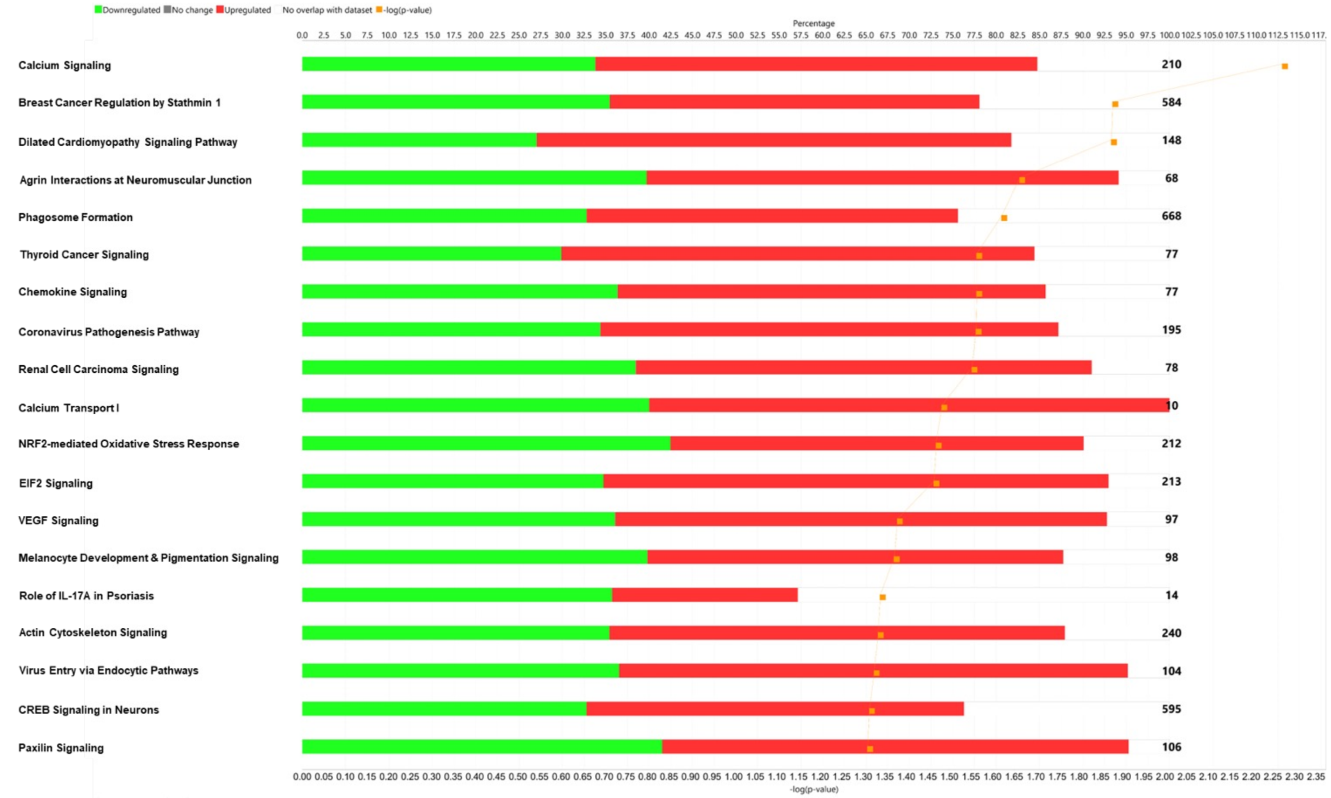

Figure S8 Signalling pathways altered by SSX3 overexpression

OVCAR-3 cells overexpressing SSX3 or a vector control plasmid were analysed by RNA-Seq to identify how the presence of SSX3 cDNA impacts global gene expression. DE genes between SSX3 and the control group were identified using cut off values of ± 1.5 fold change and *p* value = ≤0.05. IPA software was used to analyse the canonical signalling and metabolic pathways affected by the DE genes in the dataset. The orange bar indicated -log(p-value), green bar indicates percentage of downregulated genes and red bar indicates upregulated genes. The total number of genes involved in each pathway is highlighted in bold at the end of the bar.

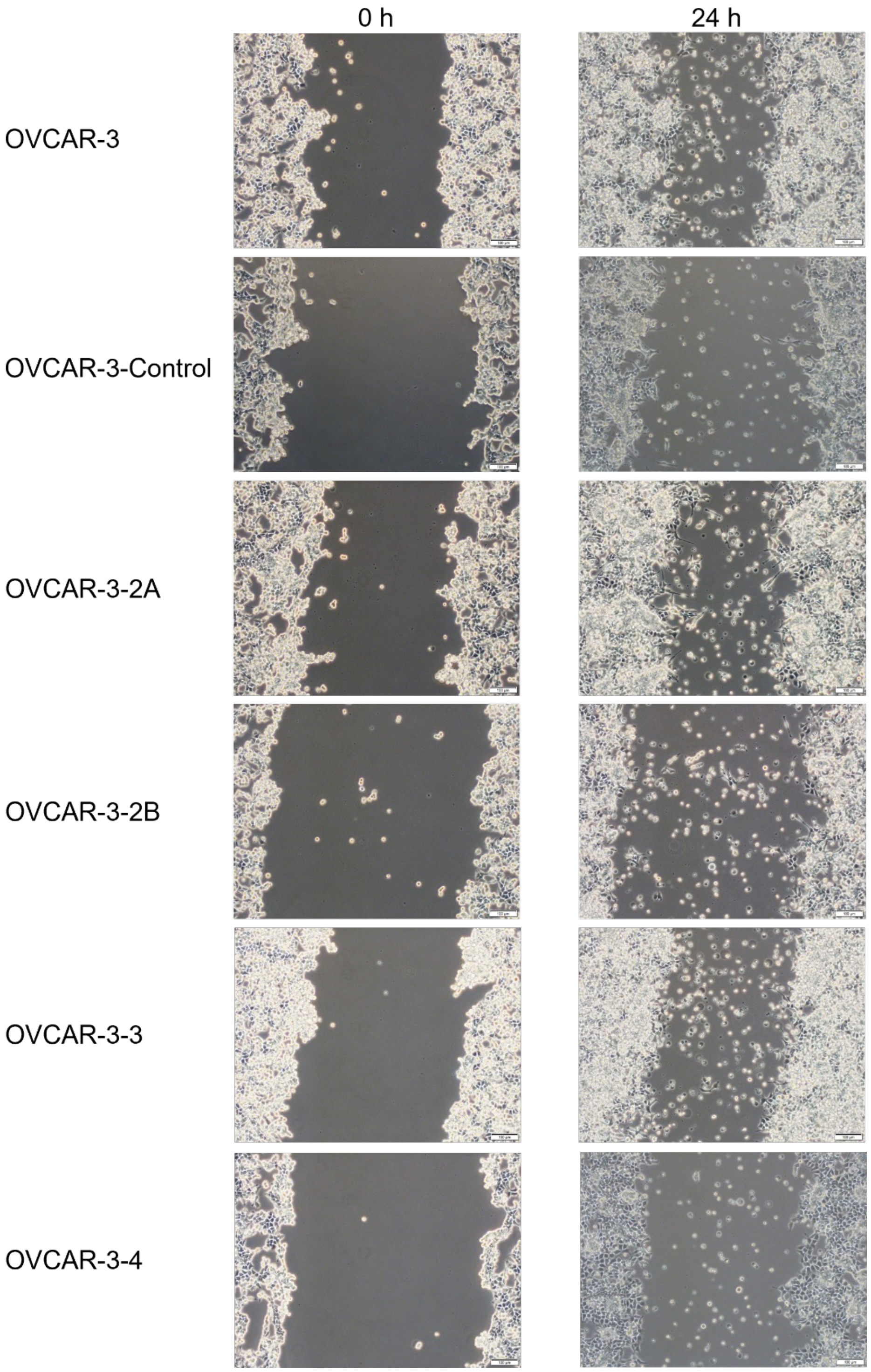

Figure S9 Effect of SSX overexpression on OVCAR-3 cell migration

The OVCAR-3 cell line was transfected with SSX2A, SSX2B, SSX3, SSX4 or a vector ‘control’ plasmid. Once cells had formed a confluent monolayer a scratch was performed. Using a Zeiss Axio Inverted Fluorescence microscope, pictures are representative of wound closure immediately and 24 hours (h) after wounding. Scale bar = 100 µm, magnification 10X.

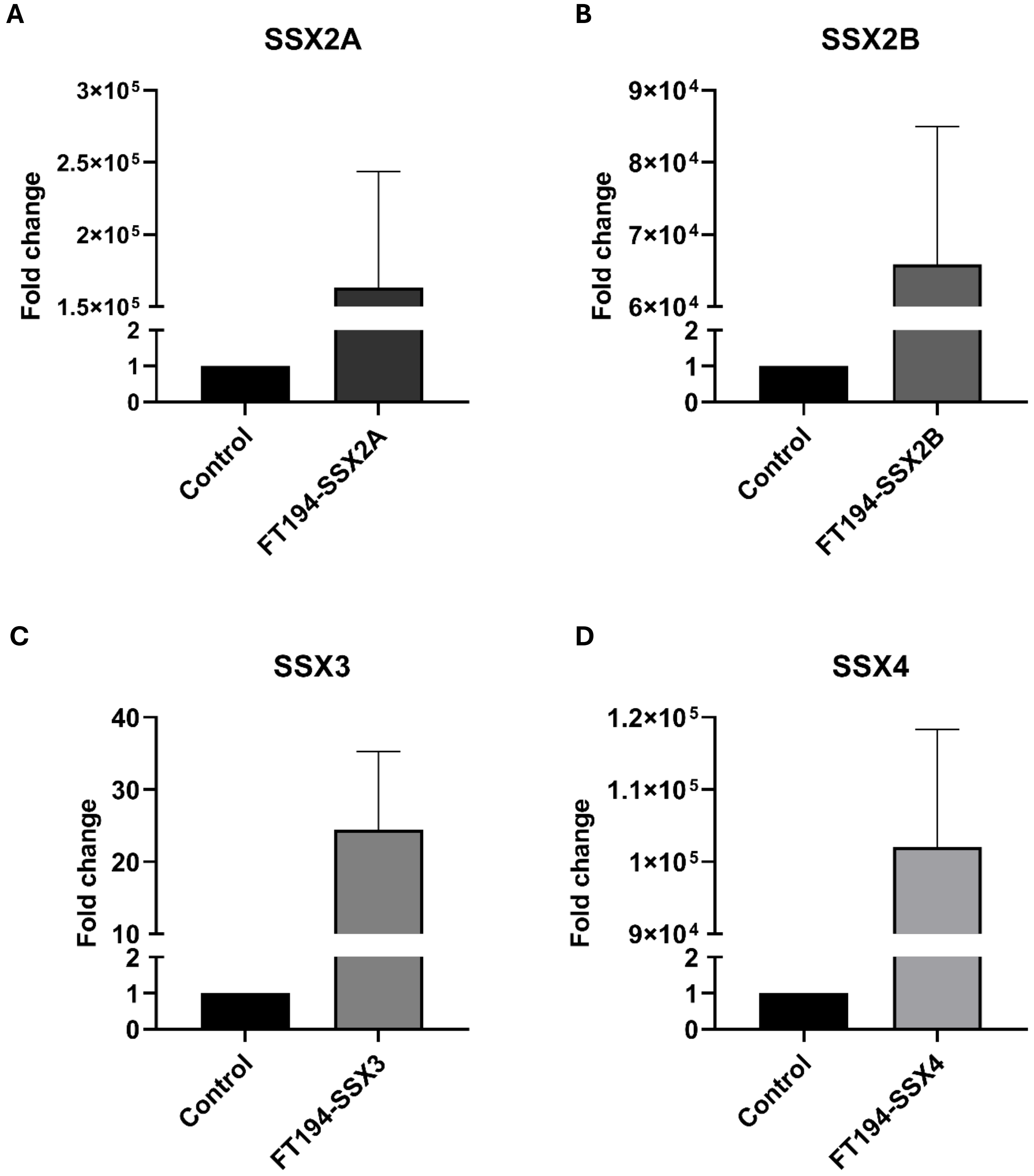

Figure S10 Confirmation of FT94 transient transfection with SSX2A, SSX2B, SSX3 and SSX4 expressing plasmids by qPCR

The fallopian tube cell line FT194 was transiently transfected with SSX2A, SSX2B, SSX3, SSX4 or the vector 'control' plasmid, and samples were collected 18 hours post-transfection. Successful transfection of FT194 was confirmed by determining the transcript expression of (A) SSX2A, (B) SSX2B, (C) SSX3 or (D) SSX4 relative to expression in the control using qPCR. The fold change was calculated relative to the vector control using the $2^{-\Delta\Delta CT}$ method. GAPDH was used as the endogenous reference gene. Data representative of *n* = 3 experimental repeats, error bars ± SEM.
