# Supplementary material for: Distinct and Convergent Roles of the Synovial Sarcoma X Breakpoint Family of Cancer-Testis Antigens in Ovarian Cancer
Source: Int J Mol Sci. 2026 Sep 26;27(19):8608. doi: 10.3390/ijms27198608 (PMC13665215; doi:10.3390/ijms27198608)
Supplement: Supplementary file 1 [file ijms-27-08608-s001.zip › Supplemental Tables.pdf]

Table S1 Cell lines

Cell line	Origin	Cell type	Culture type	Growth media	Supplements
A2780	ENOC	Epithelial	Adherent	RPMI	10% FBS
FT194	Fallopian tube	Epithelial	Adherent	DMEM/F12	10% FBS
HeLa	Cervical adenocarcinoma	Epithelial	Adherent	DMEM	10% FBS
K562	Chronic myeloid leukaemia	Lymphoblast	Non-adherent	DMEM	10% FBS
OVCAR-3	OC ascites	Epithelial	Adherent	RPMI	20% FBS 0.01 mg/ml bovine insulin
SKOV-3	OC	Epithelial	Adherent	McCoys 5A Medium Modified	10% FBS
Abbreviations	DMEM: Dulbecco's Modified Eagle Medium; ENOC: endometrioid ovarian cancer; RPMI: Roswell Park Memorial Institute				

Table S2 QuantiTECT primer assays used for real-time quantitative PCR

Target	Amplicon length (bp)	GeneGlobe ID
ACTC1	105	QT00205296
ARL17B	81	QT01662437
COMMD6	150	QT00070357
E-cadherin (CDH1)	84	QT00080143
GAPDH	95	QT00079247
HE4	131	QT00001659
ISX	163	QT00011396
PNMA3	110	QT00249200
PTK2	150	QT00057687
RGPD8	117	QT01663949
SSX1	106	QT00051779
SSX2	140	QT00042385
SSX2B	140	QT01335936
SSX3	96	QT00036722
SSX4	142	QT00068719
TMEM27	113	QT00022309
TNNT2	139/136	QT00089782
Abbreviations	ACTC1, Actin Alpha Cardiac Muscle 1; ARL17B, Adenosine Diphosphate Ribosylation Factor Like GTPase 17B; bp, base pairs; COMMD6, copper metabolism gene MURR1 Domain Containing 6; GAPDH, Glyceraldehyde-3-Phosphate Dehydrogenase; ISX, Intestine Specific Homeobox; ITGA6, Integrin Subunit Alpha 6; PNMA3, Paraneoplastic Ma Antigen 3; PTK2, Protein Tyrosine Kinase 2; RGPD8, RAN Binding Protein 2 Like And GRIP Domain Containing 8; TMEM27, Transmembrane protein 27; TNNT2, Troponin T2 Cardiac Type; ZEB-2, Zinc Finger E-Box Binding Homeobox 2	

Table S3 Primary antibodies

Target	Clone	Manufacturer
β-Actin	ACTN05	Fisher
Ki67	Ab15580	Abcam
SSX1	OTI2E9	Sigma-Aldrich
SSX2	Ab172209	Abcam
SSX2 (4D10)	GTX83555	Genetex
SSX2 (C-terminal)	Ab48751	Abcam
SSX2 (N-terminal)	Ab182361	Abcam
SSX3	SAB1406948	Sigma-Aldrich
SSX4	Ab58011	Abcam
SSX5	Ab126225	Abcam
SSX6	Ab83385	Abcam

Table S4 Top upregulated and downregulated genes in response to SSX2A overexpression

Gene name	Full name	Fold change	*p*-value
**Upregulated**			
NACAD	NAC alpha domain containing	22.70	0.014
TNNT2	Troponin T2, cardiac type	20.03	6.4×10^{-3}
NUGGC	Nuclear GTPase, germinal center associated	17.61	0.036
NEUROG2	Neurogenin 2	16.84	0.017
SMIM11A	Small integral membrane protein 11	4.67	1.6×10^{-4}
TNFRSF8	TNF receptor superfamily member 8	4.03	0.042
ADRB2	Adrenoceptor beta 2	3.34	0.015
RNF224	Ring finger protein 224	3.33	0.046
ENTPD8	Ectonucleoside triphosphate diphosphohydrolase 8	2.27	0.035
GRIK5	Glutamate ionotropic receptor kainate type subunit 5	2.17	0.025
**Downregulated**			
HRH4	Histamine Receptor H4	-24.24	0.012
AC010327.1	-	-20.46	0.024
CGB8	Chorionic Gonadotropin Subunit Beta 8	-20.25	0.041
OTOP2	Otopetrin 2	-19.68	0.027
LRRTM2	Leucine Rich Repeat Transmembrane Neuronal 2	-18.91	0.031
FAM24A	Family With Sequence Similarity 24 Member A	-18.91	0.031
HIST1H2BG	H2B Clustered Histone 8	-18.37	0.040
ALDOB	Aldolase, fructose-bisphosphate B	-17.17	0.013
TBX15	T-Box Transcription Factor 15	-15.40	0.019
ACTC1	Actin Alpha Cardiac Muscle 1	-12.47	0.038

Table S5 Top upregulated and downregulated genes in response to SSX2B overexpression

Gene name	Full name	Fold change	*p*-value
**Upregulated**			
ASB4	Ankyrin Repeat and SOCS Box Containing 4	15.10	0.022
ADAMTS12	ADAM Metallopeptidase with Thrombospondin Type 1 Motif 12	13.17	0.030
SERF1A	Small EDRK-Rich Factor 1A	13.05	$5.5x10^{-3}$
NEUROG2	Neurogenin 2	11.80	0.041
SERF1B	Small EDRK-Rich Factor 1B	4.08	0.014
RGS16	Regulator Of G Protein Signalling 16	1.31	0.028
ZNF22	Zinc Finger Protein 22	1.25	0.041
LIG3	DNA ligase 3	1.19	0.027
PCDH7	Protocadherin 7	1.15	0.039
**Downregulated**			
CLTRN	Collectrin	-21.86	0.013
AL133352.1	-	-21.34	0.016
CFAP54	Cilia And Flagella Associated Protein 54	-19.21	0.027
PLA2G12B	Phospholipase A2 Group XIIB	-17.91	0.034
OTOP2	Otopetrin 2	-17.18	0.033
RGPD4	RANBP2 Like and GRIP Domain Containing 4	-15.67	0.047
GCM1	Glial Cells Missing Transcription Factor 1	-14.15	0.023
TBX15	T-Box Transcription Factor 15	-13.44	0.025
CATIP	Ciliogenesis Associated TTC17 Interacting Protein	-12.47	0.033
MAGEB3	Melanoma-Associated antigen B3	-11.40	$1.8x10^{-3}$

Table S6 Top upregulated and downregulated genes in response to SSX3 overexpression

Gene name	Full name	Fold change	*p*-value
**Upregulated**			
GCHFR	GTP cyclohydrolase I feedback regulator	26.11	0.023
FGD2	FYVE, RhoGEF and PH domain-containing protein 2	23.70	0.018
PNMA3	Paraneoplastic antigen MA	22.57	0.019
ISX	Intestine Specific Homeobox	21.83	0.028
CD27	Cluster of differentiation 27	17.66	0.028
SHISA7	Shisa Family Member 7	16.02	0.025
TNNT2	Troponin T2, Cardiac Type	12.63	0.046
PIP5K1B	Phosphatidylinositol-4-phosphate 5-kinase type 1 beta	9.25	0.043
ERAS	ES Cell Expressed Ras	7.55	0.039
BOLA2B	BolA Family Member 2	6.70	0.021
**Downregulated**			
ACTC1	Actin alpha cardiac muscle 1	-18.70	0.027
STXBP5L	Syntaxin Binding Protein 5L	-9.11	0.043
FNDC5	Fibronectin type III domain containing 5	-7.01	0.023
SARNP	SAP Domain Containing Ribonucleoprotein	-6.98	7.4×10^{-3}
RNF148	Ring Finger Protein 148	-6.94	0.026
HACD4	3-hydroxyacyl-CoA dehydratase 4	-6.33	0.042
PKD2L2	Polycystin 2 Like 2, Transient Receptor Potential Cation Channel	-6.02	0.027
PHOSPHO2	Phosphatase, Orphan 2	-5.98	1×10^{-3}
LOX	Lysyl Oxidase	-4.98	0.040
TMEM217	Transmembrane Protein 217	-4.55	9.1×10^{-3}

Table S7 Genes upregulated and downregulated in response to SSX2A and SSX2B overexpression

Gene name	Full name	SSX2A		SSX2B	
		Fold change	*p*-value	Fold change	*p*-value
**Upregulated**					
**NEUROG2**	Neurogenin 2	16.84	0.017	11.80	0.041
**Downregulated**					
**ADCY5**	Adenylate Cyclase 5	-2.78	0.014	-2.33	0.039
**ALB**	Albumin	-5.07	0.031	-4.57	0.037
**C1S**	Complement C1s subcomponent	-2.07	0.038	-1.99	0.041
**CLEC18B**	C-Type Lectin Domain Family 18 Member B	-2.58	0.020	-2.43	0.034
**GPR179**	G Protein-Coupled Receptor 179	-5.34	1.9×10^{-3}	-2.49	0.044
**HIST1H2AC**	H2A Clustered Histone 6	-1.64	6.9×10^{-3}	-1.49	0.015
**HIST1H4K**	H4 Clustered Histone 12	-3.38	0.024	-2.64	0.044
**MAGEB3**	Melanoma-Associated Antigen Family Member B3	-4.06	0.016	-11.40	1.8×10^{-3}
**OTOP2**	Otopetrin 2	-19.68	0.027	-17.18	0.033
**REELD1**	Reeler Domain Containing 1	-3.01	0.047	-6.80	4.3×10^{-3}
**RSPH4A**	Radial Spoke Head Component 4A	-2.81	0.011	-2.31	0.029
**TBX15**	T-Box Transcription Factor 15	-15.40	0.019	-13.44	0.025

Table S8 Genes upregulated and downregulated in response to SSX2A and SSX3 overexpression

Gene name	Full name	SSX2A		SSX3	
		Fold change	*p*-value	Fold change	*p*-value
**Upregulated**					
**AC008687.4**	-	1.98	0.039	2.18	0.027
**C19orf71**	Chromosome 19 Open Reading Frame 71	1.76	6.2×10^{-3}	1.72	0.041
**KDM8**	Lysine Demethylase 8	1.67	0.041	1.76	0.030
**TNFRSF8**	TNF Receptor Superfamily Member 8	4.03	0.042	4.60	0.026
**TNNT2**	Troponin T2, Cardiac Type	20.03	6.4×10^{-3}	12.63	0.046
**Downregulated**					
**ACTC1**	Actin Alpha Cardiac Muscle 1	-12.47	0.038	-18.70	0.027
**ATP6V0C**	ATPase H+ Transporting V0 Subunit C	-1.63	4×10^{-3}	-2.05	0.022
**C11orf71**	Chromosome 11 Open Reading Frame 71	-2.07	0.011	-2.15	0.014
**FNDC5**	Fibronectin type III domain containing 5	-7.39	0.019	-7.01	0.023
**RPS15A**	Ribosomal Protein S15a	-1.36	0.022	-1.45	0.022
**SARNP**	SAP Domain Containing Ribonucleoprotein	-3.65	0.011	-6.98	7.4×10^{-3}
**SHLD3**	Shieldin Complex Subunit 3	-1.73	0.023	-2.26	9×10^{-3}
**STXBP5L**	Syntaxin Binding Protein 5L	-10.65	0.023	-9.11	0.043

Table S9 Genes upregulated and downregulated in response to SSX2B and SSX3 overexpression

Gene name	Full name	SSX2B		SSX3	
		Fold change	*p*-value	Fold change	*p*-value
**Upregulated**					
**CEL**	Carboxyl Ester Lipase	2.82	7.6×10^{-3}	2.46	0.044
**Downregulated**					
**GPR89B**	G Protein-Coupled Receptor 89B	-1.68	0.023	-1.92	0.015

Table S10 Three genes were downregulated in response to SSX2A, SSX2B and SSX3 overexpression

Gene name	Full name	SSX2A		SSX2B		SSX3	
		Fold change	*p*-value	Fold change	*p*-value	Fold change	*p*-value
**ARL17B**	ADP Ribosylation Factor Like GTPase 17B	-2.41	0.018	-2.37	0.022	-2.40	0.043
**COMMD6**	Copper metabolism gene MURR1 Domain-Containing Protein 6	-1.59	1.2×10^{-4}	-1.39	0.033	-1.53	0.048
**RGPD8**	RAN Binding Protein 2 Like and GRIP Domain Containing 8	-1.74	6.8×10^{-3}	-1.85	1.1×10^{-3}	-2.26	2.5×10^{-3}

Table S11 Two functional clusters of genes with altered expression were identified in the DE genes following SSX2A overexpression in OVCAR-3 cells

Gene	Full name
**Cluster 1**	
**H2AC6**	H2A clustered histone 6
**H3C6**	H3 clustered histone 6
**H2BC5**	H2B clustered histone 5
**H4C12**	H4 clustered histone 12
**H2BC8**	H2B clustered histone 8
**Cluster 2**	
**SLC22A17**	Solute carrier family 22 member 17
**FNDC5**	Fibronectin type III domain containing 5
**PKDREJ**	Polycystin family receptor for egg jelly
**ADRB2**	Adrenoceptor beta 2
**CRHR2**	Corticotropin releasing hormone receptor 2
**SMIM11**	Small integral membrane protein 11
**ZACN**	Zinc activated ion channel
**GPR179**	G protein-coupled receptor 179
**REELD1**	Reeler domain containing 1
**OR51M1**	Olfactory receptor family 51 subfamily M member 1
**ST7L**	Suppression of tumorigenicity 7 like
**OTOP2**	Otopetrin 2
**HRH4**	Histamine receptor H4
**TNFRSF8**	TNF receptor superfamily member 8

Table S12 List of the most significant pathways identified through Reactome following SSX2A overexpression

Term	Gene count	*p* value	Bonferroni Šidák *p* value	Genes
Transcriptional regulation of granulopoiesis	6	1.6×10^{-5}	5.9×10^{-3}	E2F1, H2AC6, H2BC5, H2BC8, H3C6, H4C12
Pre-NOTCH Transcription and Translation	6	1.8×10^{-5}	6.9×10^{-3}	E2F1, H2AC6, H2BC5, H2BC8, H3C6, H4C12
Pre-NOTCH Expression and Processing	6	3.9×10^{-5}	1.5×10^{-2}	E2F1, H2AC6, H2BC5, H2BC8, H3C6, H4C12
RNA Polymerase I Promoter Opening	5	7.0×10^{-5}	2.6×10^{-2}	H2AC6, H2BC5, H2BC8, H3C6, H4C12
Oxidative Stress Induced Senescence	6	7.6×10^{-5}	2.8×10^{-2}	E2F1, H2AC6, H2BC5, H2BC8, H3C6, H4C12
DNA methylation	5	8.0×10^{-5}	3.0×10^{-2}	H2AC6, H2BC5, H2BC8, H3C6, H4C12
Activated PKN1 stimulates transcription of androgen receptor regulated genes KLK2 and KLK3	5	9.0×10^{-5}	3.4×10^{-2}	H2AC6, H2BC5, H2BC8, H3C6, H4C12
RUNX1 regulates transcription of genes involved in differentiation of HSCs	6	9.1×10^{-5}	3.4×10^{-2}	GATA3, H2AC6, H2BC5, H2BC8, H3C6, H4C12
SIRT1 negatively regulates rRNA expression	5	9.5×10^{-5}	3.6×10^{-2}	H2AC6, H2BC5, H2BC8, H3C6, H4C12
Assembly of the ORC complex at the origin of replication	5	1.0×10^{-4}	3.8×10^{-2}	H2AC6, H2BC5, H2BC8, H3C6, H4C12
Abbreviations	HSCs, hematopoietic stem cells; KLK, kallikrein; ORC, origin recognition; PKN1, protein kinase N1; RUNX1, runt-related transcription factor 1; SIRT1, sirtuin 1			

Table S13 Functional clusters using the DE genes following SSX2B overexpression in OVCAR-3 cells

Gene	Full name
**Cluster 1**	
**SLC31A2**	Solute carrier family 31 member 2
**CFAP54**	Cilia and flagella associated protein 54
**OTOP2**	Otopetrin 2
**REELD1**	Reeler domain containing 1
**TMEM185A**	Transmembrane protein 185A
**TMEM42**	Transmembrane protein 42
**Cluster 2**	
**NEUROG2**	Neurogenin 2
**GCM1**	Glial cells missing transcription factor 1
**ZNF691**	Zinc finger protein 691
**TBX15**	T-box transcription factor 15
**ZSCAN30**	Zinc finger and SCAN domain containing 30
**HOXB6**	Homeobox B6
**ZNF22**	Zinc finger protein 22

Table S14 List of the most significant pathways identified through Reactome following SSX2B overexpression

Term	Gene count	*p* value	Bonferroni Šidák *p* value	Genes
Meiotic synapsis	4	1.0×10^{-3}	0.24	H2AC6, H2BC11, H4C12, STAG3
Base Excision Repair	4	1.6×10^{-3}	0.34	LIG3, H2AC6, H2BC11, H4C12
Meiosis	4	3.3×10^{-3}	0.57	H2AC6, H2BC11, H4C12, STAG3
Human Reproduction	4	5.6×10^{-3}	0.77	H2AC6, H2BC11, H4C12, STAG3
Packaging Of Telomere Ends	3	7.7×10^{-3}	0.87	H2AC6, H2BC11, H4C12
Depurination	3	8.9×10^{-3}	0.90	H2AC6, H2BC11, H4C12
Cleavage of the damaged purine	3	8.9×10^{-3}	0.90	H2AC6, H2BC11, H4C12
Recognition and association of DNA glycosylase with site containing an affected purine	3	8.9×10^{-3}	0.90	H2AC6, H2BC11, H4C12
Depyrimidination	3	1.1×10^{-2}	0.90	H2AC6, H2BC11, H4C12
Cleavage of the damaged pyrimidine	3	1.1×10^{-2}	0.90	H2AC6, H2BC11, H4C12

Table S15 Functional cluster resulting from the DE genes following SSX3 overexpression in OVCAR-3 cells

Gene	Full name
**TMEM126B**	Transmembrane protein 126B
**FNDC5**	Fibronectin type III domain containing 5
**NPIPB13**	Nuclear pore complex interacting protein family, member B13
**TMEM254**	Transmembrane protein 254
**PKD2L2**	Polycystin 2 like 2, transient receptor potential cation channel
**GPR89B**	G protein-coupled receptor 89B
**TMEM234**	Transmembrane protein 234
**FAM156A**	Family with sequence similarity 156 member A
**TMEM126A**	Transmembrane protein 126A
**SHISA7**	Shisa family member 7
**NPIPB3**	Nuclear pore complex interacting protein family member B3
**TMEM238**	Transmembrane protein 238
**TMEM217**	Transmembrane protein 217
**RNF148**	Ring finger protein 148
**TNFRSF8**	TNF receptor superfamily member 8

Table S16 List of the statistically significant pathways identified through Reactome following SSX3 overexpression

Term	Gene count	*p* value	Genes
**Cellular responses to stress**	7	3.7×10^{-2}	ATP6V0C, CXCL8, DNAJB9, MDM2, RPS15A, RPS28, UBC
**SARS-CoV-1-host interactions**	3	3.8×10^{-2}	RPS15A, RPS28, UBC
**Cellular responses to stimuli**	7	4.0×10^{-2}	ATP6V0C, CXCL8, DNAJB9, MDM2, RPS15A, RPS28, UBC
**TNFR2 non-canonical NF-kB pathway**	3	4.2×10^{-2}	CD27, TNFRSF8, UBC
**SRP-dependent cotranslational protein targeting to membrane**	3	5.0×10^{-2}	RPS15A, RPS28, SRP19
Abbreviations	SARS-CoV-1, severe acute respiratory syndrome coronavirus 1; SRP, signal recognition particle; TNFR2, Tumour necrosis factor receptor type II		
